# *OLIGOCELLULA1*/*HIGH EXPRESSION OF OSMOTICALLY RESPONSIVE GENES15* Promotes Cell Proliferation With *HISTONE DEACETYLASE9* and *POWERDRESS* During Leaf Development in *Arabidopsis thaliana*

**DOI:** 10.3389/fpls.2018.00580

**Published:** 2018-05-03

**Authors:** Marina Suzuki, Nanae Shinozuka, Tomohiro Hirakata, Miyuki T. Nakata, Taku Demura, Hirokazu Tsukaya, Gorou Horiguchi

**Affiliations:** ^1^Department of Life Science, College of Science, Rikkyo University, Tokyo, Japan; ^2^Research Center for Life Science, College of Science, Rikkyo University, Tokyo, Japan; ^3^Graduate School of Biological Sciences, Nara Institute of Science and Technology, Nara, Japan; ^4^Graduate School of Science, The University of Tokyo, Tokyo, Japan; ^5^Okazaki Institute for Integrative Bioscience, Okazaki, Japan

**Keywords:** Arabidopsis, OLI1, HOS15, HDA9, PWR, leaf size, cell proliferation, N-CoR/SMRT

## Abstract

Organ size regulation is dependent on the precise spatial and temporal regulation of cell proliferation and cell expansion. A number of transcription factors have been identified that play a key role in the determination of aerial lateral organ size, but their functional relationship to various chromatin modifiers has not been well understood. To understand how leaf size is regulated, we previously isolated the *oligocellula1* (*oli1*) mutant of *Arabidopsis thaliana* that develops smaller first leaves than the wild type (WT) mainly due to a reduction in the cell number. In this study, we further characterized *oli1* leaf phenotypes and identified the *OLI1* gene as well as interaction partners of OLI1. Detailed characterizations of leaf development suggested that the cell proliferation rate in *oli1* leaf primordia is lower than that in the WT. In addition, *oli1* was associated with a slight delay of the progression from the juvenile to adult phases of leaf traits. A classical map-based approach demonstrated that *OLI1* is identical to *HIGH EXPRESSION OF OSMOTICALLY RESPONSIVE GENES15* (*HOS15*). *HOS15*/*OLI1* encodes a homolog of human transducin β-like protein1 (TBL1). TBL1 forms a transcriptional repression complex with the histone deacetylase (HDAC) HDAC3 and either nuclear receptor co-repressor (N-CoR) or silencing mediator for retinoic acid and thyroid receptor (SMRT). We found that mutations in *HISTONE DEACETYLASE9* (*HDA9*) and a switching-defective protein 3, adaptor 2, N-CoR, and transcription factor IIIB-domain protein gene, *POWERDRESS* (*PWR*), showed a small-leaf phenotype similar to *oli1*. In addition, *hda9* and *pwr* did not further enhance the *oli1* small-leaf phenotype, suggesting that these three genes act in the same pathway. Yeast two-hybrid assays suggested physical interactions, wherein PWR probably bridges HOS15/OLI1 and HDA9. Earlier studies suggested the roles of HOS15, HDA9, and PWR in transcriptional repression. Consistently, transcriptome analyses showed several genes commonly upregulated in the three mutants. From these findings, we propose a possibility that HOS15/OLI1, PWR, and HDA9 form an evolutionary conserved transcription repression complex that plays a positive role in the regulation of final leaf size.

## Introduction

Various organs in multicellular organisms reach a final size that fulfills their specific functions and that is determined by complex developmental programs governing cell proliferation and expansion. The leaf is an ideal organ to investigate the mechanisms of organ size regulation owing to its determinate growth (Donnelly et al., 1999; Ichihashi et al., 2011; Andriankaja et al., 2012). A leaf primordium is initiated at the flank of the shoot apical meristem. During the early phases of leaf development, active cell proliferation occurs throughout the leaf primordium followed by cell differentiation that initiates from the tip of the leaf primordium and is associated with a rapid increase in cell volume. Cell proliferation in the leaf primordia is supported by at least four distinct meristematic activities – i.e., marginal meristem, plate meristem, leaf meristem, and meristemoids (White, 2006; Tsukaya, 2017). A zone of transition from cell proliferation to expansion, called the arrest front (Nath et al., 2003; Ichihashi et al., 2011), is maintained for a while and eventually disappears (Kazama et al., 2010; Andriankaja et al., 2012). After the cessation of cell expansion in the entire leaf primordium, leaf morphogenesis is completed (Donnelly et al., 1999). Genetic, molecular, and biochemical analyses of *Arabidopsis thaliana* (hereafter, Arabidopsis) have identified numerous genes that influence leaf size through modulating cell proliferation and/or cell expansion activities (Tsukaya, 2013; Hepworth and Lenhard, 2014). Forward genetic approaches have contributed to the identification of regulatory components that are involved in cell proliferation and cell expansion (Horiguchi et al., 2006a,b; Pérez-Pérez et al., 2009). This trend was followed by identification of the functional connection between them from the molecular to organ levels (Kalve et al., 2014; Rodriguez et al., 2014; Ichihashi and Tsukaya, 2015). Eventually, understanding how the activities of these regulatory factors are translated into cellular behaviors to determine the final organ size should be a major goal of this topic (González and Inzé, 2015; Ichihashi and Tsukaya, 2015), and such knowledge should contribute to the improvement of agricultural traits (Che et al., 2015; Duan et al., 2015; Gao et al., 2015).

Transcriptional regulation has emerged as a key process in organ size regulation. For example, AINTEGUMENTA (ANT)/ANT-LIKE (AIL), GROWTH REGULATING FACTOR (GRF), and ANGUSTIFOLIA3/GRF-INTERACTING FACTOR (AN3/GIF) families positively regulate cell proliferation in leaf primordia (Mizukami and Fischer, 2000; Kim and Kende, 2004; Horiguchi et al., 2005; Nole-Wilson et al., 2005; Lee et al., 2009; Rodriguez et al., 2010). On the other hand, class II TEOSINTE BRANCHED1/CYCLOIDEA/PROLIFERATING CELL NUCLEAR ANTIGEN FACTOR (TCP), PEAPOD (PPD), NGATHA (NGA), and SQUAMOSA PROMOTER BINDING-LIKE (SPL) families, and SPATULA (SPT) negatively regulate this process (Nath et al., 2003; Palatnik et al., 2003; White, 2006; Usami et al., 2009; Ichihashi et al., 2010; Lee et al., 2015; Zhang et al., 2015; Alvarez et al., 2016). In addition, 20 transcription factors form a network to regulate cell proliferation in response to mild osmotic stress (Van den Broeck et al., 2017).

An important role of a transcription factor is to recruit a protein complex that enables chromatin modification and/or remodeling to activate or repress its target gene expression and induce a specific developmental output. AN3/GIF and GRF form a transcription factor complex. In addition, recent tandem affinity purification (TAP) experiments identified the constituents of the AN3/GRF complex, including the chromatin-remodeling ATPases BRAHMA (BRM) and SPLAYED (Vercruyssen et al., 2014; Kim and Tsukaya, 2015). TCP4 also interacts with BRM, binds the promoter region of *ARABIDOPSIS RESPONSE REGULATOR16*, and induces its expression to repress cytokinin responses (Efroni et al., 2013). Another TAP experiment has demonstrated that PPD interacts with KINASE-INDUCIBLE DOMAIN (KIX8) and KIX9 to recruit the co-repressor TOPLESS (TPL) (Gonzalez et al., 2015). The protein complex containing PPD and KIX8/9 directly represses *CYCD3* family members (Gonzalez et al., 2015).

Transcriptional activation and repression in eukaryotes are regulated by various histone modifications and their removal. Histone deacetylation is carried out by histone deacetylases (HDACs), typically resulting in gene repression (Liu et al., 2014). The Arabidopsis genome contains three distinct HDAC families (Pandey et al., 2002; Hollender and Liu, 2008; Alinsug et al., 2009). Members of the type I HDAC family belong to the Reduced potassium dependency 3 (Rpd3)-like superfamily which is widely conserved in eukaryotes. These HDACs are further divided into three classes (Pandey et al., 2002; Hollender and Liu, 2008). On the other hand, the type II HDAC family represents a plant-specific family. The type III HDAC family is homologous to Silent information regulator 2 (Sir2) and is also widely conserved in eukaryotes. While some of these HDACs are known to regulate specific developmental processes such as flowering and body axis formation (Long et al., 2006; Liu et al., 2014), none of them have known functions in terms of leaf-size regulation.

Histone deacetylases function by forming a protein complex with co-repressors, transcription factors, and other adapter and accessory proteins to repress specific target genes (Liu et al., 2014). The Groucho (Gro)/dTMP-Uptake1 (Tup1)-like group of the WD40 repeat protein family is the best characterized co-repressor family and contains at least 13 members in Arabidopsis. This group includes TPL, four TPL-RELATEDs (TPRs), LEUNIG (LUG), LEUNIG_HOMOLOG (LUH), HIGH EXPRESSION OF OSMOTICALLY RESPONSIVE GENES15 (HOS15), and several uncharacterized members (Liu and Karmarkar, 2008). TPL/TPR members were identified from *tpl* in which the embryo produces an ectopic root instead of the shoot apical meristem (Long et al., 2006). Later studies demonstrated that TPL/TPRs bind the ethylene response factor-associated amphiphilic repression (EAR) motif that is found in a number of transcription repressors and adapter proteins (Causier et al., 2012; Ke et al., 2015; Martin-Arevalillo et al., 2017). LUG, together with its interactor SEUSS (SEU), represses the floral homeotic gene *AGAMOUS* and regulates floral organ identity (Conner and Liu, 2000; Franks et al., 2002). LUG also plays a negative role in leaf size regulation through cell expansion (Cnops et al., 2004). LUG and its homolog LUH form a complex with SEU, SEU-LIKE, and YABBY, and maintain leaf polarity and the activity of the shoot apical meristem (Stahle et al., 2009). On the other hand, HOS15 is involved in offsetting the expression levels of stress-responsive genes (Zhu et al., 2008). For HOS15, neither its interacting proteins nor developmental roles have been described. Thus, current understandings concerning the relationship between Gro/Tup1-like proteins and organ size regulation are also very limited.

We previously isolated a number of mutants with an altered leaf size and classified them according to changes in cell number and size. The *oligocellula* (*oli*) class of mutants has a specific cell proliferation defect without strongly affecting cell size (Horiguchi et al., 2006a,b). Among the members of this class, *OLI2* encodes a putative m5C methyltransferase for rRNA, while *OLI5* and *OLI7* encode ribosomal proteins (RPL5A and RPL5B), suggesting an important role for ribosome biogenesis and function in cell proliferation in leaf primordia (Fujikura et al., 2009; Kojima et al., 2018). To better understand leaf size regulation, the identification of responsible genes in the remaining *oli* mutants and their functional characterization is necessary. In this study, we identified *HOS15* as the causal gene of *oli1*. A previous report showed that the loss of function of *HOS15* increases the acetylated histone H4 level and hyperactivation of stress-responsive genes such as *RD29A* under stress conditions (Zhu et al., 2008), but did not characterize developmental phenotypes. HOS15 is closely related to transducin β-like protein1 (TBL1) in humans. TBL1 forms a complex with HDAC3 (a member of the Rpd3-like HDAC family) and either nuclear receptor co-repressor (N-CoR) or silencing mediator for retinoic acid and thyroid receptor (SMRT) (Guenther et al., 2000; Li et al., 2000). Both N-CoR and SMRT have two switching-defective protein 3, adaptor 2, N-CoR, and transcription factor IIIB (SANT) domains that are flanked by long stretches of intrinsically disordered regions (Watson et al., 2012). In Arabidopsis, no apparent homolog(s) of N-CoR/SMRT has been identified, and it has been suggested that components of the protein complex containing HOS15, if any, would not share a high level of sequence similarity with N-CoR/SMRT (Zhu et al., 2008). On the other hand, a physical interaction between a SANT domain-containing protein POWERDRESS (PWR) and HDA9 was recently reported in Arabidopsis (Chen et al., 2016; Kim et al., 2016). However, whether PWR and HDA9 form a protein complex with a TBL1-like protein in Arabidopsis is not known. Here, we carried out genetic and molecular analyses of OLI1/HOS15, HDA9, and PWR, and show that these three proteins act in the same pathway, probably by functioning as a protein complex to promote cell proliferation in leaf primordia.

## Materials and Methods

### Plant Materials

The wild-type (WT) accession used in this study was Columbia-0. The isolation of *oli1-1* was previously reported (Horiguchi et al., 2006a,b; Fujikura et al., 2009). The alleles of *hda6* [*auxin gene expression 1–5* (*axe1–5*) (Murfett et al., 2001)], *hda7* [Salk_002912 (Cigliano et al., 2013)], *hda9* [Salk_007123/*hda9-1* and GABI_305G03/*hda9-2* (Kim et al., 2013)], *pwr* [Salk_071811/*pwr-2* (Yumul et al., 2013), Salk_006823/*pwr-10*], and *mir156c* [Salk_004679 (Yu et al., 2013)] were obtained from the Arabidopsis Biological Resource Center. The seeds of *kluh-4* (*klu-4*) kindly provided by Michael Lenhard (University of Potsdam) were sown on rock wool covered by pulverized peat moss and grown at 22°C under a photoperiod of 16 h of light/8 h of darkness. Nutrient solution [0.5 g L^-1^ Hyponex (Hyponex Japan, Osaka, Japan)] was supplied daily.

### Quantitative Analyses of Leaf Development

To determine the leaf blade size, as well as the size and number of adaxial subepidermal cells, mature first leaves (21–25 days after sowing) were fixed in formalin–acetic acid–alcohol and were cleared in chloral hydrate solution as described in Horiguchi et al. (2005). Next, the samples were subjected to stereoscopic microscopy (M165FC; Leica, Wetzlar, Germany) and differential interference contrast microscopy (DM2500; Leica, Wetzlar, Germany). To determine the number of adaxial epidermal pavement cells, the first leaves of 21-day-old seedlings were frozen in liquid nitrogen and were immediately observed with a scanning electron microscope (JCM-6000; JEOL, Tokyo, Japan). The cell proliferation rate in leaf primordia was determined as described by De Veylder et al. (2001).

### Genetic Mapping

To prepare a mapping population, *oli1-1* and Landsberg *erecta* were crossed, and F2 seeds were sown on rockwool. Segregants showing the small-leaf phenotype of *oli1* were selected, and their genomic DNA was extracted. The *OLI1* locus was mapped using polymorphic markers according to the sequence information available at The Arabidopsis Information Resource (TAIR^1^). The nucleotide sequences of the candidate region of the *OLI1* locus were determined to identify the *oli1-1* mutation point.

### Generation of Transgenic Plants

To generate transgenic *oli1-1* plants carrying a p*35S*::*HOS15* construct, *HOS15* cDNA was amplified and cloned into pENTR/D-TOPO (Thermo Fisher Scientific, Waltham, MA, United States). Then *HOS15* cDNA was transferred into a binary vector, pH35G (Horiguchi et al., 2005). The *HOS15* cDNA was also transferred into pH35GW (G. Horiguchi and H. Tsukaya, unpublished data) to express GFP-HOS15. Genomic DNA fragments containing approximately 300 bp upstream of the initiator codon of *HOS15* and uidA cDNA for β-glucuronidase (GUS) were amplified and cloned into pENTR P4P1R and pDONR201 (Thermo Fisher Scientific, Waltham, MA, United States), respectively, by BP clonase II (Thermo Fisher Scientific, Waltham, MA, United States), and they were combined into the binary vector pGWB501 (Nakagawa et al., 2007) by LR clonase II plus (Thermo Fisher Scientific, Waltham, MA, United States). The resultant construct was introduced into WT plants to produce p*HOS15*::*GUS* transgenic lines. To generate p*CYCB1;1*:*GUS* reporter transgenic plants, an approximately 1.2-kb promoter region plus a partially transcribed region of *CYCB1;1* was amplified so that the destruction box of CYCB1;1 was translationally fused with GUS (Donnelly et al., 1999). The amplified genomic DNA fragment was cloned into pSMAB704 (kindly provided by H. Ichikawa), which was pre-digested with HindIII and SmaI using the In-Fusion HD Cloning Kit (Takara Bio, Shiga, Japan).

To prepare an RNA interference (RNAi) construct, two *HOS15* cDNA fragments flanked by SmaI/HindIII and SacI/NotI, respectively, were cloned into pENTI (Horiguchi et al., 2005), and its inverted-repeat region was transferred into pH35G by LR clonase II (Thermo Fisher Scientific, Waltham, MA, United States) (Horiguchi et al., 2005). An approximately 800-bp promoter plus transcribed region lacking the termination codon of *PWR* was amplified and cloned into pENTR/D-TOPO (Thermo Fisher Scientific, Waltham, MA, United States) and transferred into pHWG (Kojima et al., 2018) by LR clonase II to express *PWR-GFP* under the control of its own promoter in the *pwr-2* background. An approximately 900-bp promoter plus the transcribed region of *HDA9* lacking the termination codon, *Venus* cDNA, and an approximately 2.5-kb region downstream of the *HDA9* termination codon were amplified by PCR and combined into pSMAB704 predigested by HindIII and EcoRI using the In-Fusion HD Cloning Kit (Takara Bio, Shiga, Japan). The nucleotide sequences of primers used to construct these vectors are listed in Supplementary Table S1.

Transformation of Arabidopsis plants was carried out by the floral dip method (Clough and Bent, 1998). At least two independent transgenic plants with a single T-DNA insertion were established, and their homozygous lines were analyzed at the T3 generation except p*PWR*::*PWR-GFP*/*pwr-2* and p*HDA9*::*HDA9-Venus*/*hda9-1* plants, which were examined at the T2 generation.

### RNA Isolation and Expression Analyses

Total RNA was isolated from the shoots grown for an indicated period and was subjected to reverse transcription. The total RNA was extracted using Trizol reagent (Thermo Fisher Scientific, Waltham, MA, United States) according to the manufacturer’s instructions. Reverse transcription-quantitative PCR (RT-qPCR) was carried out using SuperScript III (Thermo Fisher Scientific, Waltham, MA, United States), followed by GoTaq qPCR master mix (Promega, Madison, WI, United States) with a 7500-Fast Real-Time PCR System (Thermo Fisher Scientific, Waltham, MA, United States). The expression levels of the genes of interest were normalized by the ΔΔCT method with *ACTIN2* (*ACT2*) as the control gene. For semi-quantitative RT-PCR, the cDNAs were amplified by Blend Taq (Toyobo, Osaka, Japan). The primer pairs used in these expression analyses are listed in Supplementary Table S1 except that those for *SPL* genes were described in Usami et al. (2009). For microarray analysis, the total RNA was isolated using first and second leaf primordia harvested from 8-day-old WT and *hos15-2* seedlings grown on rockwool. The isolated RNAs were subjected to transcriptome analysis with the Agilent Arabidopsis oligo DNA microarray Ver.4.0 (Agilent) by Miltenyi Biotec (Tokyo, Japan). The *q*-values of false-discovery rate (FDR) were calculated with BH method (Benjamini and Hochberg, 1995) using the R Stats package^2^ (R Core Team, 2017).

### Yeast Two-Hybrid Assay

The yeast two-hybrid (Y2H) assay was carried out using the ProQuest Two-Hybrid System (Thermo Fisher Scientific, Waltham, MA, United States). The cDNAs of *HOS15, HDA9*, and *PWR* were amplified and cloned into pENTR/D-TOPO, and the inserted cDNAs were transferred into pDEST22 and pDEST32 by LR clonase II. The resultant fusion constructs were used to transform yeast cells. The transformed cells were cultured in synthetic complete medium lacking leucine, tryptophan, and histidine, but containing 20 mM 3-amino-1,2-4-triazole to test the occurrence of protein–protein interactions. The primer sequences used in the construction of the Y2H vectors are listed in Supplementary Table S1.

### Bimolecular Fluorescence Complementation (BiFC) Assay

The cDNAs of *HOS15, HDA9*, and *PWR* with or without the termination codon were cloned into pENTR/D-TOPO and transferred to a series of vectors for the BiFC assay (Tanaka et al., 2012). These constructs were introduced into leaf mesophyll protoplasts according to Yoo et al. (2007). The transfected protoplasts were observed using a confocal laser scanning microscope (LSM800; Carl Zeiss, Oberkochen, Germany). The primer sequences used in the construction of the BiFC vectors are listed in Supplementary Table S1.

### Other Microscopic Observations

Histochemical GUS staining was carried out according to Donnelly et al. (1999). For GUS staining, the seedlings were grown on rockwool or half-strength Murashige and Skoog (MS) medium supplemented with 3% (w/v) sucrose. The seedlings that expressed either *PWR-GFP* or *Venus-HDA9* were grown on rockwool or half-strength MS medium supplemented with 3% (w/v) sucrose and were fixed and cleared using ClearSee (Kurihara et al., 2015). For GFP-HOS15 observation, seedlings were fixed, stained with 4′1,6-diamidino-2-phenylindole (DAPI), and cleared according to Ohtani et al. (2013). The cleared tissues were observed using a confocal laser scanning microscope (LSM800, Carl Zeiss, Oberkochen, Germany).

## Results

### Developmental Defects of *oli1*

**FIGURE 1:**
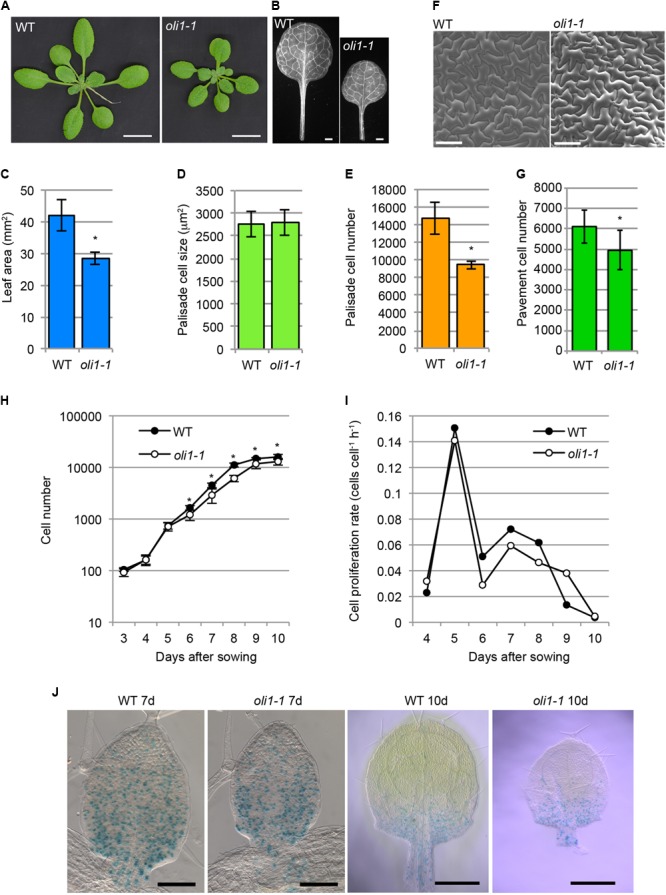
Characterizations of the leaf phenotypes in *oli1-1*. **(A)** Shoots of wild-type (WT) and *oli1-1*. **(B)** First leaves of WT and *oli1-1*. **(C)** The areas of the first leaf blades. **(D,E)** Quantitative phenotypes of palisade cells in the adaxial subepidermal layer (hereafter called palisade cells for simplicity) of the first leaves of WT and *oli1-1*. The projection areas of palisade cells of the first leaves **(D)**, and estimated palisade cell numbers **(E)** are shown. **(F)** Adaxial epidermis in the first leaves of WT and *oli1-1* observed under a scanning electron microscope. **(G)** Pavement cell numbers in the adaxial epidermal tissue first leaves. **(H,I)** Time course analysis of first leaf development. Changes in the cell number **(H)** and cell proliferation rate **(I)** were determined by observing the palisade cell layers of first leaf primordia at the indicated days after sowing. **(J)** Expression pattern of p*CYCB1;1*::*GUS* in WT and *oli1-1* grown for 7 (left two panels) and 10 days (right two panels). Scale bars in **(A)** and **(B)** correspond to 1 cm and 1 mm, respectively, those in **(F)** and the left two panels in **(J)** are 100 μm, and those in the right two panels in **(J)** are 0.5 mm. In **(A–G)**, the samples were harvested at 21 days after sowing. In **(C–H)**, the data are shown as means ± SD (*n* = 10), and asterisks indicate statistically significant differences between WT and *oli1-1* (Student’s *t*-test, *p* < 0.05). In **(I)**, the data were calculated from those shown in **(H)**.

We previously reported that the number of palisade cells in the subepidermal layer (hereafter, palisade cells, for simplicity) of the first leaves with *oli1-1* was reduced to approximately 60–50% of the WT level without significantly affecting the cell size and overall leaf shape (Horiguchi et al., 2006a,b; Fujikura et al., 2009; **Figures 1A–E**). The reduction in the cell number was also found in the leaf adaxial epidermis (**Figures 1F,G**), suggesting a general reduction in cell proliferation activity in *oli1-1* leaf primordia. A time course analysis of the first leaf development suggested that *oli1* had a slightly lower cell proliferation rate than WT, and this difference became evident beyond 6 days after seed sowing (**Figures 1H,I**). We crossed a p*CYCB1;1*::*GUS* reporter line with *oli1-1* to visualize cells at the G2/M phase. However, we could not find a clear difference in the distribution of the GUS signals in leaf primordia and timing of the disappearance of the GUS signals (**Figure 1J**).

**FIGURE 2:**
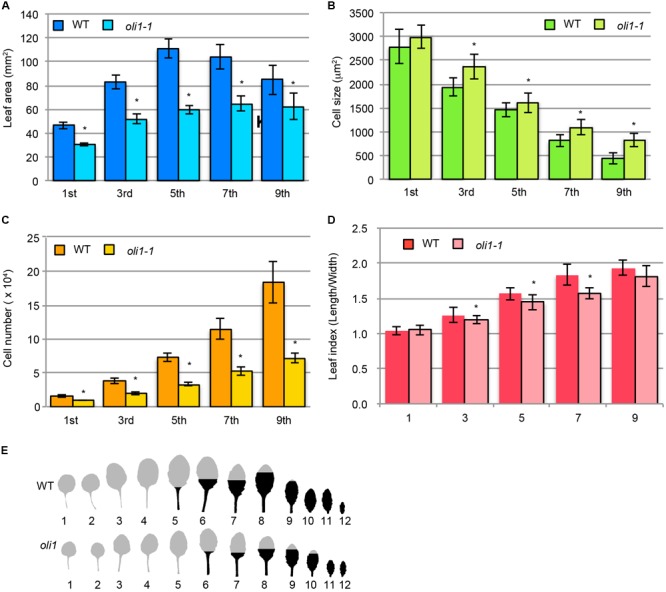
Characterization of leaf phenotypes during the transition from the juvenile to adult phases in *oli1-1*. **(A)** Leaf blade areas. **(B)** Projection areas of palisade cells. **(C)** Estimated palisade cell numbers. **(D)** Leaf index (the ratio of leaf blade length to width). The data are shown as means ± SD (*n* = 10), and asterisks indicate statistically significant differences between WT and *oli1-1* (Student’s *t*-test; *p* < 0.05). **(E)** Distribution of abaxial trichomes. A portion of the leaf blades is filled in black to indicate the upper limit of the abaxial trichome distribution. The trichome distribution was observed using 31-day-old plants. In **(A–D)**, the first and third, fifth and seventh, and ninth leaves were harvested at 24, 27, and 31 days after sowing, respectively. In **(A–E)**, the leaf positions were numbered from the oldest leaves on the bottom row of each panel.

Next, we characterized leaf development in terms of heteroblasty, a phenomenon in which several leaf traits change along with the progression of plant age from the juvenile to adult phases (Poethig, 2013). The leaf size and cell number and size in different leaves change in association with heteroblasty (Usami et al., 2009). The largest leaves in WT were the fifth leaves, but were the sixth or seventh leaves in *oli1-1* (**Figures 2A,E**). The leaf blade areas of *oli1-1* leaves were always smaller than those of WT irrespective of the leaf positions (**Figure 2A**). In WT, palisade cells progressively increased their number, but decreased their size as leaves are formed in the more adult phase (**Figures 2B,C**). These trends were also found in *oli1*, but the number and size of palisade cells were fewer and larger in *oli1* leaves than in WT at any of the leaf positions examined (**Figures 2B,C**). At the same time, the leaf index (the ratio of the leaf blade length to width) also increased progressively in later formed leaves (Tsukaya et al., 2000). Compared with the WT leaves, the leaf indexes of the third, fifth, and seventh leaves of *oli1-1* were slightly smaller than the corresponding leaves of WT (**Figure 2D**). Because these changes in the leaf phenotypes suggest a delayed progression of the juvenile to adult phase transition, we examined trichome distribution on abaxial epidermal tissues. Trichomes are absent from on the abaxial side of juvenile blades, but they begin to form during the transition into adult leaves from the basal part of leaf blades. Adult leaves have abaxial trichomes even in their uppermost part (Telfer et al., 1997). In WT, abaxial trichomes first appeared in the fifth leaves; however, in *oli1-1*, they did so in the sixth leaves (**Figure 2E**). The observed results shown in **Figure 2** suggest that *oli1-1* may slightly delay the progression of heteroblasty compared with that the WT plants.

In contrast to the clear leaf phenotypes, the lengths of the primary roots in *oli1-1* were only slightly longer than those in the WT (Supplementary Figure S1A). On the other hand, the sizes of the flowers in WT and *oli1-1* were similar to each other (Supplementary Figure S1B).

### Identification of the *OLI1* Gene

**FIGURE 3:**
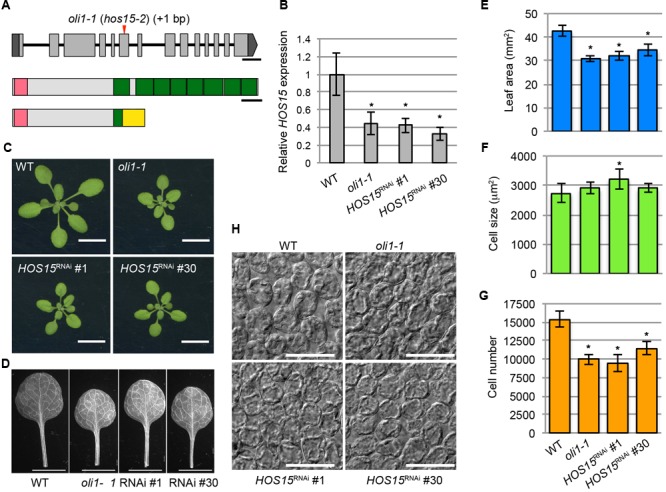
Identification of the causal gene of the *oli1-1* mutation. **(A)** Schematic diagrams of the *OLI1*/*HOS15* locus (top) and HOS15 protein (middle) and the deduced structure of mutated oli1-1 protein (bottom). The mutation point of *oli1-1* (renamed *hos15-2*) is indicated by a red arrowhead. Exons and introns are indicated by boxes and lines, respectively. The 5′- and 3′-untranslated regions of *OLI1*/*HOS15* are indicated in dark gray, while the coding regions are indicated in light gray. The amino-terminal LisH motifs are indicated by pink boxes, while the carboxy-terminal WD40 repeats are indicated by green boxes. A yellow box indicates the region generated due to the frame shift by the *oli1-1* mutation. Bars indicate 300 bp and 50 amino acids, respectively. **(B–G)** Characterization of *HOS15* knockdown plants by RNAi (*HOS15*^RNAi^ No. 1 and No. 30). **(B)** Expression levels of *HOS15* determined by RT-qPCR using 10-day-old shoots. Data are shown as means ± SD. Asterisks indicate statistically significant differences compared with the WT value (*n* = 3, Student’s *t*-test, *p* < 0.05). **(C)** Shoots. **(D)** First leaves. **(E)** Areas of the first leaves. **(F)** Projection area of the palisade cells. **(G)** Estimated palisade cell numbers. **(H)** Palisade cells observed from the paradermal view. In **(C–H)**, 21-day-old plants were used. In **(E–G)**, the data are shown as means ± SD (*n* = 10), and the asterisks indicate significant differences compared with the WT values (Student’s *t*-test; *p* < 0.05). The bars in **(C), (D)**, and **(H)** indicate 1 cm, 5 mm, and 100 μm, respectively.

The chromosomal position of the *OLI1* locus was determined by classical genetic mapping within an approximately 68-kb region of the lower end of chromosome 5 where 14 genes were found (Supplementary Figure S2). Sequencing of this region identified a single base insertion in the sixth exon of *HOS15* that caused a frame-shift and created a premature termination codon (**Figure 3A**). *HOS15* encodes a WD40 protein implicated in the transcriptional repression of stress-responsive genes (Zhu et al., 2008). It has an LisH motif in the amino terminus and eight WD40 repeats in the carboxy-terminal side (**Figure 3A**). The predicted open-reading frame of *oli1-1* encoded a carboxy terminally truncated protein that had an incomplete first WD40 repeat and lacked subsequent repeats (**Figure 3A**). To examine whether *HOS15* was the causal gene of *oli1-1*, we generated two RNAi lines of *HOS15* (*HOS15*^RNAi^ No. 1 and No. 30). These lines decreased the expression levels of *HOS15* to about 35–40% of the WT level (**Figure 3B**). In *oli1-1, HOS15* expression was also decreased to a similar level found in the RNAi lines (**Figure 3B**). Consistently, the two RNAi lines produced small shoots and rosette leaves (**Figures 3C–E**) and were associated with a decrease in the palisade cell number, but the palisade cells were relatively normal in size (**Figures 3F–H**). Occasionally, the palisade cell sizes in *oli1-1* and *HOS15*^RNAi^ lines were larger than in WT in different trials (an example can be seen in *HOS15*^RNAi^ No. 1 shown in **Figure 3F**). A similar increase in the leaf cell size has been often observed in mutants strongly defective in cell proliferation and is known as compensated cell enlargement (Horiguchi and Tsukaya, 2011; Hisanaga et al., 2015). Whether this is a compensation phenotype should be interpreted carefully because a stronger delay in the progression of heteroblasty might make leaves with fewer but larger palisade cells. Indeed, the youngest leaves – i.e., cotyledons – have fewer but larger palisade cells than the first leaves (Ferjani et al., 2007). We also confirmed that overexpression of *HOS15* in the *oli1-1* background rescued the *oli1-1* leaf phenotypes (Supplementary Figure S3). Together, these results demonstrated that *OLI1* corresponds to *HOS15*. Thus, we renamed *oli1-1* as *hos15-2*.

### *HOS15, HDA9*, and *PWR* Act in the Same Genetic Pathway to Positively Regulate Leaf Size

HOS15 is a member of the Gro/Tup1-like WD40 proteins and, thus, is expected to function with an Rpd3-like HDAC(s) (Guenther et al., 2000; Li et al., 2000). We first focused on class I Rpd3-like HDACs, HDA6, HDA7, and HDA9 (Hollender and Liu, 2008; Alinsug et al., 2009). HDA19 is also a member of this class, but was excluded from our analysis because it is known to function in a complex containing TPL/TPR (e.g., Krogan et al., 2012; Ryu et al., 2014; Pi et al., 2015). Amino acid sequences of HDA6 and HDA7 have 48.9 and 47.8% similarities to HDA9 judging from multiple sequence alignments by ClustalW^3^. When mutants for these genes were examined, *hda9-1* showed a small-leaf phenotype (Supplementary Figures S4C,E,H). *axe1-5* carries a point mutation at the junction of exon 3/intron 3 of *HDA6* (Murfett et al., 2001; Supplementary Figure S4A). In *axe1-5, HDA6* transcripts were abnormally spliced (Murfett et al., 2001) and we also detected abnormally spliced *HDA6* transcripts with a significant reduction of normally spliced products (Supplementary Figure S4G). However, we did not found clear reduction in leaf size (Supplementary Figure S4H). A T-DNA insertion mutant, Salk_002912, overexpressed *HDA7* (Cigliano et al., 2013) and we also confirmed this result (Supplementary Figures S4B,D,F), but it did not noticeably affect the leaf size (Supplementary Figure S4H). Although these observations did not necessarily ruled out a possibility that HDA6 and HDA7 have a role in HOS15-dependent leaf-size regulation, these results prompted us to focus on HDA9 as a strong candidate protein that functions in close association with HOS15. Next, we examined the loss-of-function phenotypes of *hda9-1* and *hda9-2* (Kim et al., 2013; Supplementary Figure S5). Both alleles showed *hos15*-like phenotypes; they produced smaller leaves with a smaller number of palisade cells (Supplementary Figures S5A,B,D). Interestingly, the size of the palisade cells in *hda9* was clearly larger than that in WT (Supplementary Figures S5C,E).

Given that HOS15 is a homolog of TBL1 and it interacts with N-CoR/SMRT (Guenther et al., 2000; Li et al., 2000), we next hypothesized that HOS15, HDA9, and the Arabidopsis homolog of N-CoR/SMRT, if any, act in the same complex. A BLASTP search using the human SMRT sequence as a query found an Arabidopsis protein encoded by *PWR* that has locally limited similarities to SMRT (Supplementary Figures S6A,B). The similar regions corresponded to two SANT domains of SMRT (Supplementary Figures S6A,B). In addition, both SMRT and PWR have short, scattered stretches of low-complexity sequences, although there were no detectable sequence similarities outside the SANT domains (Supplementary Figures S6A,B). Very recently, PWR was shown to interact with HDA9 (Chen et al., 2016; Kim et al., 2016). Thus, we characterized *pwr-2* (Yumul et al., 2013) and an additional T-DNA insertion allele (named *pwr-10*) of *pwr* (Supplementary Figures S6C,D). RT-PCR analysis using two primers that were positioned upstream and downstream of the T-DNA insertion site of each allele failed to amplify the expected *PWR* cDNA fragments showing that these two alleles did not accumulate intact *PWR* transcripts (Supplementary Figures S6E). Both alleles accumulated *PWR* mRNA fragments corresponding to the 5′-region at a level comparable to that of WT. On the other hand, 3′-*PWR* mRNA fragments were accumulated at a reduced and an undetectable level in *pwr-2* and *pwr-10*, respectively (Supplementary Figure S6E). These results suggest that these two T-DNA insertion mutants are strong alleles. These two *pwr* mutants also produced small leaves with a reduced number of palisade cells that were larger than those in WT, a phenotype similar to that of *oli1* and almost identical to that of *hda9* (Supplementary Figures S6F–K). Initially, *pwr* was named after its bulged carpel tip (Yumul et al., 2013), and this phenotype was also found in *hda9* (Kim et al., 2016) and *hos15-2* (Supplementary Figure S7).

**FIGURE 4:**
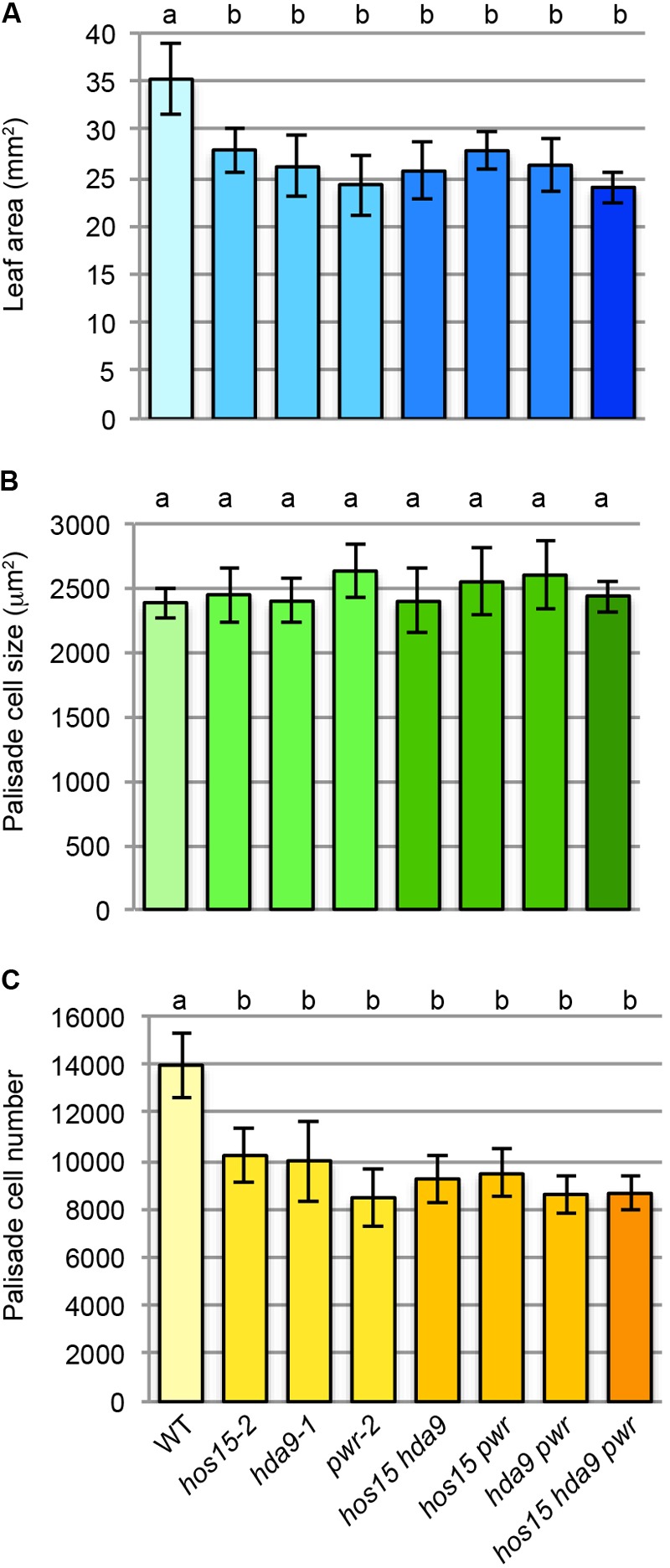
Multiple mutant analyses among *hos15-2, hda9-1*, and *pwr-2*. **(A)** Leaf area. **(B)** Palisade cell size. **(C)** Palisade cell number. The first leaves of 25-day-old leaves were used for the quantitative analyses (*n* = 9–11). Statistical analyses were carried out by one-way ANOVA using the Tukey–Kramer *post hoc* test (*p* < 0.01). Data without a significant difference are labeled by the same letter.

The mostly identical leaf and fruit phenotypes observed among *hos15, hda9*, and *pwr* led us to examine their double- and triple-mutant phenotypes (**Figure 4**). The first leaf size and number of leaf palisade cells did not further decrease in *hos15-2 hda9-1, hos15-2 pwr-2, hda9-1 pwr-2*, or *hos15-2 hda9-1 pwr-2* compared with that in parental single mutants (**Figures 4A,C**). The palisade cell size in these mutants was not significantly different from that in WT (**Figure 4B**). The absence of enhanced leaf phenotypes in multiple mutants strongly suggests mutual dependence among HOS15, HDA9, and PWR to express their molecular function.

### Expression Patterns of *HOS15, HDA9*, and *PWR* and Subcellular Localization of Their Gene Products

**FIGURE 5:**
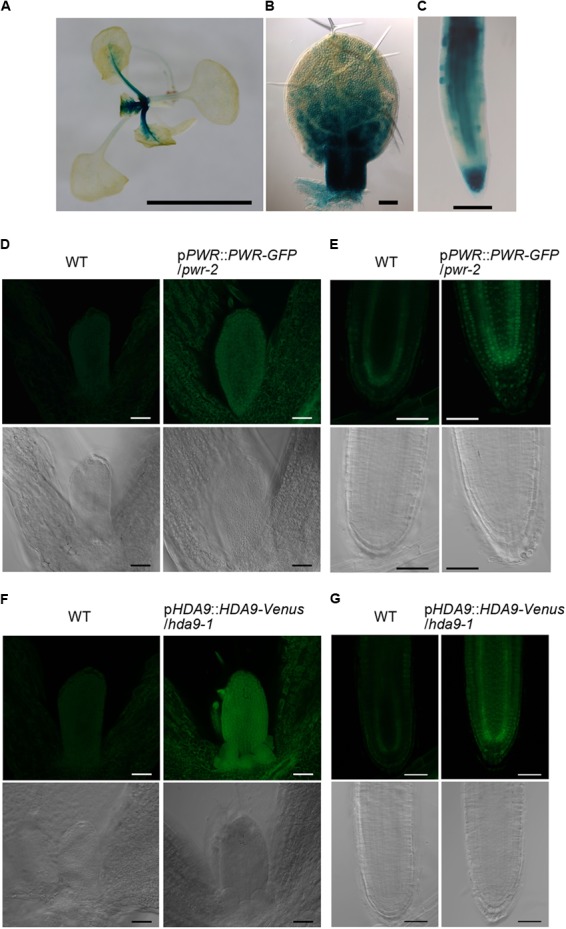
Expression patterns of *HOS15, HDA9*, and *PWR* and intracellular distribution of HDA9 and PWR. **(A–C)** Histochemical GUS staining of p*HOS15*::*GUS* plants. **(A)** A 14-day-old plant grown on rockwool. **(B)** First leaf primordium of an 8-day-old plant. **(C)** Primary root tip of a 7-day-old plant grown *in vitro*. **(D,E)** A 4-day-old p*PWR*::*PWR-GFP*/*pwr-2* plant grown *in vitro*. **(F,G)** A 4-day-old p*HDA9*::*HDA9-Venus*/*hda9-1* plant grown *in vitro*. **(D,F)** Shoot tips. **(E,G)** Root apical meristems. In **(D–G)**, WT plants were observed as a negative control and are shown in the left two panels. Images of transgenic plants carrying the *PWR-GFP* or *HDA9-Venus* construct are shown in the right two panels. Fluorescent images are shown in the upper rows while differential interference contrast images are shown in the lower rows. Bars in **(A)**, and **(D–G)** indicate 5 mm and 50 μm, respectively, while those in **(B)** and **(C)** indicate 100 μm.

The finding that *HOS15, HDA9*, and *PWR* act in the same genetic pathway strongly suggests that these three proteins act together in the same complex. If so, these genes should be expressed in the same cells or tissues. The expression of these genes in first and second leaf primordia of 8-day-old WT was detectable by RT-PCR (Supplementary Figure S8A). In addition, according to the TraVA database^4^ (Klepikova et al., 2016), the expression levels of *HOS15, HDA9*, and *PWR* in the leaf blade of third leaf primordia 3 mm in length were 2,084, 547, and 1,121 read counts (normalized by the median ratio), respectively. For comparison, the expression levels of the leaf size regulatory genes *AN3* and *ANT* were 636 and 1,524 read counts, respectively. To visualize the spatial expression pattern, we generated p*HOS15*::*GUS* lines and found strong GUS activities in developing leaf primordia and root tips (**Figures 5A–C**). In leaf primordia, strong *HOS15* expression was localized in the basal part where cell proliferation activity is high (**Figures 5A,B**). *HOS15* was also strongly expressed in the root cap and elongation zone and was weakly expressed in the internal tissues of the basal meristem (**Figure 5C**). For *PWR* and *HDA9*, we generated transgenic lines that carry a fluorescent reporter protein gene fused in the genomic context of each gene (p*PWR*::*PWR*-*GFP* and p*HDA9*::*HDA9*-*Venus*) in the respective mutant backgrounds. These lines rescued the respective small-leaf phenotypes of *pwr-2* and *hda9-1* (Supplementary Figures S8B–G). In the case of p*PWR*::*PWR-GFP*/*pwr-2* No. 11 plants, their leaf blade area and palisade cell number were larger than those of WT, suggesting that an increase in *PWR* expression could promote cell proliferation beyond the WT level. Next, we observed 4-day-old seedlings of p*PWR*::*PWR*:*GFP*/*pwr-2* and p*HDA9*::*HDA9-Venus*/*hda9-1* grown *in vitro.* Fluorescent signals of both PWR-GFP and HDA9-Venus were very weak and we observed both reporter lines along with WT plants. PWR-GFP and HDA9-Venus signals were detectable in young leaf primordia at levels clearly stronger than the autofluorescence found in the WT plants (**Figures 5D,F**). PWR-GFP and HDA9-Venus accumulated in both the cytosol and nuclei of cells in leaf primordia (**Figures 5D,F**). In root apical meristems, PWR-GFP and HDA9-Venus were concentrated in the nuclei (**Figures 5E,G**). These signals were clearly stronger than autofluorescence found in the WT cells (**Figures 5D–G**). Nuclear localization of HDA9 was also reported previously (Kang et al., 2015; Chen et al., 2016). Concerning HOS15, its nuclear localization was reported previously (Zhu et al., 2008) and we obtained the same results when a *GFP-HOS15* fusion gene was overexpressed (Supplementary Figure S9). The presence of GFP-HOS15, PWR-GFP, and HDA9-Venus in the nuclei is consistent with the expected function of these proteins in transcriptional repression.

### Protein–Protein Interactions Among HOS15, HDA9, and PWR

**FIGURE 6:**
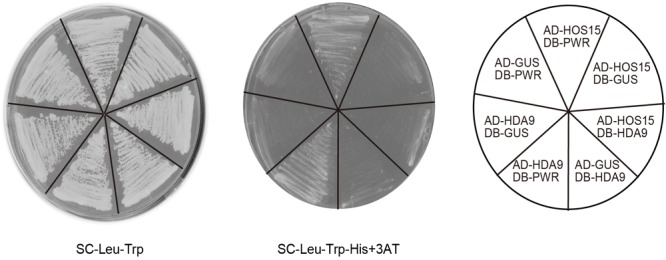
Yeast two-hybrid assay using HOS15, HDA9, and PWR. Yeast cells grown on synthetic complete (SC) medium lacking leucine (Leu) and tryptophan (Trp) are shown in the left panel. Yeast cells grown on SC lacking Leu, Trp, and histidine (His), but containing 20 mM 3-amino-1,2-4-triazole (3AT) are shown in the middle panel. Combinations of vectors used are indicated in the right panel. GUS was used as a negative control. AD and DB indicate the activation domain and DNA-binding domain of GAL4, respectively.

To examine the possibility that HOS15, HDA9, and PWR act in the same complex, we carried out Y2H assays. When HOS15 and PWR were fused with the activation domain (AD) and DNA-binding domain (DB) of GAL4, respectively, they interacted with each other (**Figure 6**). AD-HDA9 and DB-PWR also interacted with each other (**Figure 6**), consistent with the result reported by Kim et al. (2016). However, AD-HOS15 and DB-HDA9 showed no interaction (**Figure 6**). These results suggested that PWR might bridge HOS15 and HDA9 or PWR-HOS15 and PWR-HDA9 might form different complexes. To test whether HDA9 and HOS15 interact with each other, we carried out BiFC assays. However, we could not detect complemented fluorescence signals in any of the combinations of HDA9 and HOS15 fused with the C-terminal or N-terminal yellow fluorescent protein fragments (data not shown).

### Transcriptome Analysis of *hos15-2*

To understand how HOS15 regulates cell proliferation in leaves, we carried out transcriptome analysis using *hos15-2* and WT leaf primordia harvested from 8-day-old seedlings using Agilent Arabidopsis oligo DNA microarray Ver.4.0 (Supplementary Table S2). In *hos15-2*, 130 and 79 genes were upregulated and downregulated, respectively, by more than twofold compared with the WT levels (Supplementary Tables S3, S4). These genes were subjected to GO enrichment analysis (Maere et al., 2005). However, no enriched GO terms were found when examined for the GO_Biological process/molecular function/cellular component except that the GO term “transcription factor TFIID complex” was enriched (*p* < 0.05) including only two genes (At1g27720 and At3g19040) as upregulated genes.

**FIGURE 7:**
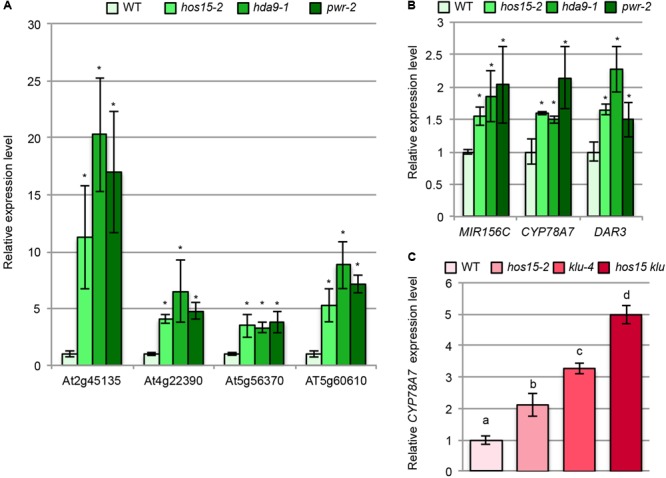
Expression analyses of the putative downstream genes of HOS15, HDA9, and PWR. **(A,B)** Total RNA from the shoots of 10-day-old WT, *hos15-2, hda9-1*, and *pwr-2* seedlings were subjected to RT-qPCR analyses. **(C)** Total RNA from the shoots of 10-day-old WT, *hos15-2, klu-4*, and *hos15-2 klu-4* seedlings were subjected to RT-qPCR analyses. *n* = 3, mean ± SD. Statistical analyses in **(A)** and **(B)** utilized Student’s *t*-test (*p* < 0.05) while that in **(C)** utilized one-way ANOVA with Tukey’s HSD test (*p* < 0.05).

We next examined whether *hos15, pwr*, and *hda9* had commonly upregulated or downregulated genes by comparing our transcriptome data with the RNA sequencing data reported by Chen et al. (2016). Among 130 and 277 upregulated genes in *hos15-2* and in both *pwr* and *hda9*, only six commonly upregulated genes were identified (Supplementary Table S5). On the other hand, we found seven common genes among 79 and 354 downregulated genes in *hos15-2* and in both *pwr* and *hda9* (Supplementary Table S5). The very small number of commonly regulated genes in these mutants would result from the difference in the samples used in our analysis (young leaf primordia) and the difference in the RNA sequencing analyses carried out by Chen et al. (2016) who characterized *pwr* and *hda9* in relation to senescence. We then examined the expression levels of four of the six upregulated genes using 10-day-old shoot RNAs by RT-qPCR and confirmed the transcriptome data (**Figure 7A**), indicating that HOS15, PWR, and HDA9 share, at least partially, common downstream genes.

Because only a few common genes were identified from the comparison described above, we next focused on several upregulated genes in *hos15-2* based on the known functions in the regulation of cell proliferation. We noted that *MIR156C* was upregulated in *hos15-2* (Supplementary Table S4), and this trend was confirmed by RT-qPCR (**Figure 7B**). *MIR156C* encodes miR156, which targets *SPL* family members (Wu and Poethig, 2006) and plays a major role together with *MIR156A* in leaf development among other eight family members (Yang et al., 2013; Yu et al., 2013). The expression levels of *pri-MIR156A* and *pri-MIR156C* are higher than *pri-MIR156B, D, E*, and *F* (Yu et al., 2013) and both genes are expressed in leaf primordia (Yang et al., 2013). Consistently, *mir156a mir156c* double mutants produce larger leaves than WT (Yang et al., 2013; Yu et al., 2013). miR156 maintains the juvenile characteristics of leaves (Wu and Poethig, 2006), and juvenile leaves have fewer but larger leaf cells than adult leaves (Usami et al., 2009). Thus, increased *MIR156C* expression seems to be well suited for the possibility that *hos15-2* delays the progression of the juvenile to adult phases.

Interestingly, we noted that *DAR3* showed an increased expression in our transcriptome data although it was not statistically significant level (Supplementary Table S2). In addition, *DAR3* was one of upregulated genes in transcriptome analyses of *pwr* and *hda9* (Chen et al., 2016) and of *hos15* (Zhu et al., 2008). *DAR3* is a member of the *DA1*/*DAR* family and *DA1, DAR1*, and *DAR2* redundantly and negatively regulate cell proliferation (Li et al., 2008). DA1 destabilizes several positive regulators of cell proliferation thereby acting as a negative regulator of cell proliferation (Dong et al., 2017). Re-examination of *DAR3* expression by RT-qPCR indeed confirmed that it was upregulated in *hos15-2, pwr-2*, and *hda9-1* (**Figure 7B**).

In addition to *MIR156C* and *DAR3*, we found that the expression of *CYP78A7* was higher in *hos15-2* than in WT (Supplementary Table S4), and this effect was also reproducible when examined by RT-qPCR (**Figure 7B**). *CYP78A7* and one of its closely related family members *CYP78A5* (also known as *KLU*) redundantly participate in the positive regulation of cell proliferation in leaf primordia and negative regulation of plastochron length (Anastasiou et al., 2007; Wang et al., 2008). In relation to these phenotypes, *CYP78A5* plays a predominant role, while the single *cyp78a7* mutant does not have a detectable developmental phenotype (Wang et al., 2008). Therefore, HOS15, HDA9, and PWR commonly regulate several known regulatory genes of leaf cell proliferation.

### Double-Mutant Analysis Between *hos15-2* and Other Cell Proliferation Defective Mutants

To further characterize the HOS15/HDA9/PWR-dependent cell proliferation pathway, we crossed a T-DNA insertion allele of *mir156c* (Yu et al., 2013; Supplementary Figures S10A,B) with *hos15-2*. If upregulation of *MIR156C* fully accounts for the reduced cell number in *hos15-2*, a loss-of-function of *mir156c* in *hos15-2* might fully suppress the cell proliferation defect. In *mir156c*, the number of leaf palisade cells was increased by 20% compared with that in WT (Supplementary Figure S10E). A similar increase in the palisade cell number was found in *mir156c hos15-2*, but this was not statistically different from that in *hos15-2* (Supplementary Figure S10E). Similarly, the presence of *mir156c* in the *HOS15*/*HOS15* or *hos15-2*/*hos15-2* background did not affect leaf blade size and palisade cell size at a statistically significant level (Supplementary Figures S10C,D). We also examined expression levels of miR156-targeted *SPL* genes. Among them, *SPL3* and *SPL15* were downregulated while *SPL6* and *SPL10* were upregulated in *hos15-2* (Supplementary Figure S11). According to the comprehensive analysis of *SPL* family members, the expression of *SPL3, SPL9*, and *SPL13* is readily detectable in leaf primordia by *in situ* hybridization (Xu et al., 2016). In addition, *SPL15* is also detectable at a weaker level compared with the three *SPL* genes (Xu et al., 2016). On the other hand, *SPL6* and *SPL10* were barely detectable (Xu et al., 2016). Thus, the total activity of *SPL* genes may be reduced in *hos15-2*. These results suggest that the upregulation of *MIR156C* has only a partial negative effect, if any, on cell proliferation in *hos15-2*.

We also wanted to examine the double mutant phenotypes of *dar3 hos15-2*. However, double-mutant construction was impractical because the *DAR3* and *HOS15* loci were tightly linked. Unfortunately, we could not identify *dar3* mutants generated by the CRISPR/Cas9 system, and the role of *DAR3* in cell proliferation in the *hos15-2* background remained unclear.

The finding that *hos15-2* upregulates *CYP78A7* expression led us to examine its expression in *klu-4* and *hos15-2 klu-4*. Interestingly, both *hos15-2* and *klu-4* had a higher *CYP78A7* expression level than WT (**Figure 7C**). The expression level of *CYP78A7* was further enhanced in *hos15-2 klu-4* (**Figure 7C**), suggesting that *HOS15* and *KLU* act in independent pathways, but their effects converge at or upstream of *CYP78A7*. We also examined whether *hos15-2* and *klu-4* had a genetic interaction in relation to cell proliferation in leaf primordia. As expected, the effects of *hos15-2* and *klu-4* on the palisade cell number and leaf size were largely additive (Supplementary Figures S10F,H). On the other hand, the strong reduction in the palisade cell number did not enhance the large-cell phenotype in *hos15-2 klu-4* compared with that in *hos15-2* (Supplementary Figure S10G).

Because both *hos15* and *an3* have a reduced cell proliferation rate (**Figure 1I**; Lee et al., 2009; Horiguchi et al., 2011), we also generated *hos15-2 an3-4*. Again, *hos15-2 an3-4* showed an additive palisade cell proliferation defect and reduction in the leaf area (Supplementary Figures S10F,H). On the other hand, despite a more severe cell proliferation defect in *hos15-2 an3-4*, its palisade cell size was not statistically different from that in *an3-4* (Supplementary Figure S10G). These results suggest that HOS15 and AN3 function through different pathways to regulate the cell proliferation rate in leaf primordia.

## Discussion

### *HOS15, HAD9, and PWR* Contribute to the Promotion of Cell Proliferation in Leaf Primordia

In this study, we identified *HOS15* as the causal gene of *oli1-1*/*hos15-2* and demonstrated that HOS15, HDA9, and PWR function together to promote cell proliferation in leaf primordia. The initial identification of *HOS15* as a negative regulator of stress responsive genes (Zhu et al., 2008) may imply that the reduced cell proliferation in *hos15* resulted from a tradeoff between growth and the stress response. However, when the upregulated genes in *hos15-2* were compared with the gene list that includes cold-inducible genes with increased expression in *hos15* (Zhu et al., 2008), there was no overlap (data not shown). The question then arises as to how HOS15/PWR/HDA9-dependent pathway regulates cell proliferation in leaf primordia.

From a kinematic point of view, the final number of cells in a leaf can be influenced by the founder cell number, duration of cell proliferation, and size of the mitotically active zone in a leaf primordium, as well as the cell division rate (Gonzalez et al., 2012). Among these parameters, *hos15* appeared to decrease the rate of cell proliferation in leaf primordia (**Figure 1**). The cell proliferation rate can be enhanced by the overexpression of *APC10* and *CDC27a*, both of which encode a different subunit of the anaphase promoting complex (APC), and these changes result in the formation of larger leaves than those of WT plants (Rojas et al., 2009; Eloy et al., 2011). There are many leaf-size mutants and transgenic plants with an altered cell number, but relatively few examples are known in which an increased or decreased cell division rate results in a corresponding change in the final leaf size (Gonzalez et al., 2012). Except for cell cycle regulators, only four examples – DELLAs, AN3/GIF families, an F-box protein FBX92, and dual-specificity MAPK phosphatase INDOLE-3-BUTYRIC ACID-RESPONSE5 (IBR5)/TINKERBELL (TINK) – are known to regulate the leaf cell proliferation rate (Achard et al., 2009; Lee et al., 2009; Horiguchi et al., 2011; Johnson et al., 2015; Baute et al., 2017). When the activities of the former three groups of genes were modified, the expression levels of several cell cycle regulator genes were altered. In contrast to these examples, our transcriptome data did not identify known cell cycle regulators as upregulated or downregulated genes in *hos15-2* (Supplementary Tables S3, S4). Although details remained unclear, further investigation of the HOS15/PWR/HDA9-dependent pathway would reveal an additional layer of regulation of leaf cell proliferation that does not directly influence cell cycle gene expression.

We found two potential links between the HOS15/ PWR/HDA9-dependent pathway and cell proliferation. The first one is related to heteroblasty (**Figure 2**). Upregulation of *MIR156C* in *hos15-2* was correlated with the delayed transition of the juvenile to adult phase in this mutant. *mir156c* had more leaf cells than WT (Supplementary Figure S10). Although *mir156c hos15-2* seemed to have more leaf cells than *hos15-2*, the difference between them was not significant (Supplementary Figure S10). However, multiple heteroblasty-related phenotypes in *hos15-2* suggested a delayed progression of the juvenile to adult transition. In addition, there was technical difficulty in detecting a small difference in the leaf cell number at a statistically significant level. Therefore, we do not completely discard the possibility that the HOS15/PWR/HDA9-dependent pathway regulates cell proliferation through *MIR156C*.

A second link came from the expression analysis of *CYP78A7*. CYP78A5/KLU and CYP78A7 have been proposed to produce an intracellular signaling molecule (Anastasiou et al., 2007; Wang et al., 2008; Eriksson et al., 2010). *klu* has fewer leaf cells due to a shorter cell proliferation time and produces small leaves (Anastasiou et al., 2007). The small-leaf phenotype of *klu* is dramatically enhanced by *cyp78a7* (Wang et al., 2008). In this study, we found that the expression level of *CYP78A7* was increased in *klu-4* (**Figure 7**), suggesting that *CYP78A7* is subjected to feedback regulation by CYP78A5/KLU activity and that increased expression of *CYP78A7* may compensate for an otherwise severe cell proliferation defect in *klu*. Interestingly, *CYP78A7* is also upregulated in *hos15-2* (**Figure 7**). Since the effects of *klu-4* and *hos15-2* on the expression level of *CYP78A7* and cell proliferation were additive (**Figure 7** and Supplementary Figure S10), *hos15-2* may negatively affect the level of a putative CYP78A5-dependent signaling molecule or its activity. However, since HOS15 and CYP78A7/KLU regulate kinetically different processes of cell proliferation, an alternative possibility is that HOS15 directly represses the expression of *CYP78A7* rather than influences CYP78A5/KLU-dependent signaling.

Finally, we also found that the sizes of palisade cells in the first leaves of *hda9* and *pwr* are larger than that of WT (Supplementary Figures S4, S5). A less pronounced increase in the palisade cell size was occasionally observed in *hos15-2* and *HOS15*^RNAi^ lines (**Figure 3**). Because the expression levels of *HOS15* in the *hos15-2* and *HOS15*^RNAi^ lines were reduced by 60–65% of the WT level (**Figure 3**), these plants probably showed weak loss-of-function phenotypes. If so, an increased palisade cell size in *hda9* and *pwr* (Supplementary Figures S4, S5) could arise from stronger loss-of-function effects and likely reflects a stronger delay in the progression of heteroblasty. Indeed, there was a tendency that *hda9-2* and *pwr-2* had higher expression levels of *MIR156C* than *hos15-2* (**Figure 7**). This idea could explain why *hos15* failed to stimulate compensated cell expansion in *an3-4* (Supplementary Figure S10), if we assume that AN3 and HOS15 regulate leaf cell expansion through independent mechanisms.

### Molecular Functions of HOS15, HDA9, and PWR

In mammals, the WD40 repeat protein TBL1, a member of the Rpd3 HDAC HDAC3 and paired SANT domain proteins N-CoR and SMRT form a complex in which N-CoR/SMRT acts as a scaffold for the other proteins (Oberoi et al., 2011). N-CoR/SMRT-like transcriptional repression complexes seem to be evolutionarily conserved in eukaryotes including *Saccharomyces cerevisiae* (yeast) and animals. In yeast, the SET3 (suppressor of variegation, enhancer of zeste, and Trithorax) complex has a similar molecular organization as N-CoR/SMRT complexes (Pijnappel et al., 2001). Furthermore, Xenopus and Drosophila have homologs of TBL1, N-CoR/SMRT, and HDAC3 (Tsuda et al., 2002; Tomita et al., 2004; Qi et al., 2008). However, whether all three proteins act in the same complex is unclear. Recent studies by Chen et al. (2016) and Kim et al. (2016) demonstrated that HDA9 and PWR physically interact with each other and repress the expression of several genes to regulate flowering time and senescence. However, whether a protein complex containing HDA9 and PWR corresponds to a plant version of the N-CoR/SMRT-like complex was not discussed. Our genetic analyses and Y2H assays not only support the physical interaction between HDA9 and PWR, but also suggest that HOS15 is a missing link for an N-CoR/SMRT-like complex in Arabidopsis. In the Y2H assay, we could not detect an interaction between HOS15 and HDA9, but these two proteins each interacted with PWR. In the BiFC assay, we could not detect an interaction between HOS15 and HDA9, but this does not necessarily exclude the possibility that these proteins are in the same protein complex if a YFP fragment is not positioned in close association with another to reconstitute a functional YFP molecule, or that fusions with a YFP fragment abolish the ability to form a complex among HOS15, HDA9, and PWR. In addition, immunoprecipitation–mass spectrometry analyses of PWR-FLAG and HDA9-FLAG identified HOS15 as a co-purified protein, although these results were not mentioned in the report (Chen et al., 2016). These results suggest that the protein complex containing HOS15, PWR, and HDA9 is probably organized so that PWR acts as a bridge for the other two proteins similar to the N-CoR/SMRT complex (Oberoi et al., 2011).

Although we did not examine the direct molecular function of the putative HOS15–PWR–HDA9 complex, it most likely acts as a transcriptional repression complex considering the following observations. First, both HOS15 and the PWR–HDA9 complex are involved in the regulation of histone acetylation (Zhu et al., 2008; Chen et al., 2016; Kim et al., 2016). Second, *hos15, pwr*, and *hda9* exhibit nearly identical leaf phenotypes, and double and triple mutants among the three mutants did not further enhance the observed leaf phenotypes (**Figure 4**). Third, several common genes are similarly upregulated in *hos15, pwr*, and *hda9* (**Figure 7**). This comparison was made among the datasets obtained by different experimental systems (microarray or RNA sequencing) and biological materials (young shoots or mature shoots). Therefore, a more comprehensive picture of commonly regulated genes by HOS15, PWR, and HDA9 should be obtained using young leaf primordia grown under the identical condition in future. These results suggest that the putative HOS15–PWR–HDA9 complex is an evolutionarily conserved N-CoR/SMRT-like co-repressor core complex.

HDA9 has multiple developmental roles. HDA9 is upregulated during callus formation from leaf explants, and *hda9* reduces the callus formation capacity (Lee et al., 2016). It also represses seedling traits in dry seeds (van Zanten et al., 2014). Repression of flowering under a short-day condition and promotion of senescence require HDA9 (Kim et al., 2013; Chen et al., 2016). Although we did not examine the heteroblasty related phenotype in *hda9*, phenotypic similarities between *hos15-2* and *hda9* suggest a possibility that HDA9 is also involved in the promotion of heteroblasty. A common feature of these phenotypes is developmental transitions that progress gradually rather than abruptly. Chromatin immunoprecipitation-sequencing analysis demonstrated that HDA9 binds to the promoters of active genes (Chen et al., 2016). In addition, the HISTONE H3 acetylation level around the transcription start site of *AGAMOUS-LIKE19* in *hda9* increases the occupancies of RNA polymerase II (Kang et al., 2015). Similarly, *hos15* upregulates *RD29A* under stress conditions, but not under normal growth conditions (Zhu et al., 2008). Whether these processes require the putative HOS15–PWR–HDA9 complex should be examined in the future, but it is worth noting that the putative HOS15–PWR–HDA9 complex may play a role to fine-tune the expression levels of active genes during developmental transitions through histone deacetylation.

The N-CoR/SMRT repression complex interacts with various transcription factors, including unliganded nuclear hormone receptors, to mediate gene repression (Watson et al., 2012). HDA9 by itself probably cannot bind its target genes because it has no known DNA-binding motif. Similarly, PWR is not supposed to be a DNA-binding protein, but preferentially binds monomodified histone H3 (K9me1, K9me2, K9ac, S10P, T11P, and K14Ac) among the nucleosomal core histones (Kim et al., 2016). On the other hand, HOS15 binds histone H4 (Zhu et al., 2008). Therefore, the putative HOS15–PWR–HDA9 complex would require transcription factors to express its function. Indeed, the PWR–HDA9 complex is proposed to be recruited to its target genes through the direct interaction between WRKY53 and HDA9 (Chen et al., 2016). According to the TraVA database, WRKY53 expression in 3-mm third-leaf primordia was barely detectable (only 53 read counts). In addition, *WRKY53* expression is highly induced prior to leaf senescence (Hinderhofer and Zentgraf, 2001). Therefore, a transcription factor(s) other than WRKY53 likely recruits the putative HOS15–PWR–HDA9 complex to its target genes in developing leaf primordia, and the identity of such transcription factor(s) should be determined in a future study.

The Arabidopsis genome contains nearly 500 known and putative transcription repressor genes (Kagale and Rozwadowski, 2010). Among them, those containing an EAR motif are predominant. The EAR motif has a core sequences, either LxLxL or DLNxxP, and they are found in 352 and 73 proteins, respectively (Kagale and Rozwadowski, 2010). These transcription factors form protein complexes containing either TPL/TPR, SAP18, or SIN3-LIKE as a co-repressor and HDA19 as a chromatin modifier. On the other hand, a smaller number of transcription co-repressors have consensus motifs distinct from the EAR motif, such as R/KLFGV and LxLxPP motifs (Kagale and Rozwadowski, 2011). However, no interacting co-repressors and HDACs for these transcription repressors have been identified. Given that both TPL and HOS15 are classified as Gro/Tup1 family co-repressors (Liu and Karmarkar, 2008), it is tempting to speculate that HOS15 may bind transcription co-repressors in a manner similar to TPL.

The discussion above does not necessarily indicate that the putative HOS15–PWR–HDA9 complex uses a single mechanism in which it is recruited to the target genes. Many EAR motif-containing transcription repressors bind TPL/TPRs through the interaction between an LxLxL motif and a hydrophobic groove in the N-terminal part of TPL (Martin-Arevalillo et al., 2017). TBL1 also has a hydrophobic groove in its N-terminal region, but it is used to interact with N-CoR/SMRT, which, in turn, binds different interaction partners through its different domains (Oberoi et al., 2011; Watson et al., 2012). Therefore, PWR might have a similar function in the interaction with transcription regulators. In addition, HDA9 and WRKY53 directly interact with each other *in vitro* (Chen et al., 2016). Thus, the putative HOS15–PWR–HDA9 complex might have multiple platforms to interact with transcription factors.

## Author Contributions

GH, TD, and HT designed and conducted the experiments. MS, NS, TH, MTN, and GH performed the experiments. GH and HT wrote the manuscript.

## Conflict of Interest Statement

The authors declare that the research was conducted in the absence of any commercial or financial relationships that could be construed as a potential conflict of interest.

## Footnotes

1 http://www.arabidopsis.org/

2 https://www.R-project.org/

3 http://bar.utoronto.ca/ntools/cgi-bin/ntools_multiplealign_w_mview.cgi

4 http://travadb.org/
