# Supplementary material for: OLIGOCELLULA1/HIGH EXPRESSION OF OSMOTICALLY RESPONSIVE GENES15 Promotes Cell Proliferation With HISTONE DEACETYLASE9 and POWERDRESS During Leaf Development in Arabidopsis thaliana
Source: Front Plant Sci. 2018 May 3;9:580. doi: 10.3389/fpls.2018.00580 (PMC5943563; doi:10.3389/fpls.2018.00580)
Supplement: Supplementary file 6 [file Presentation_1.PDF]

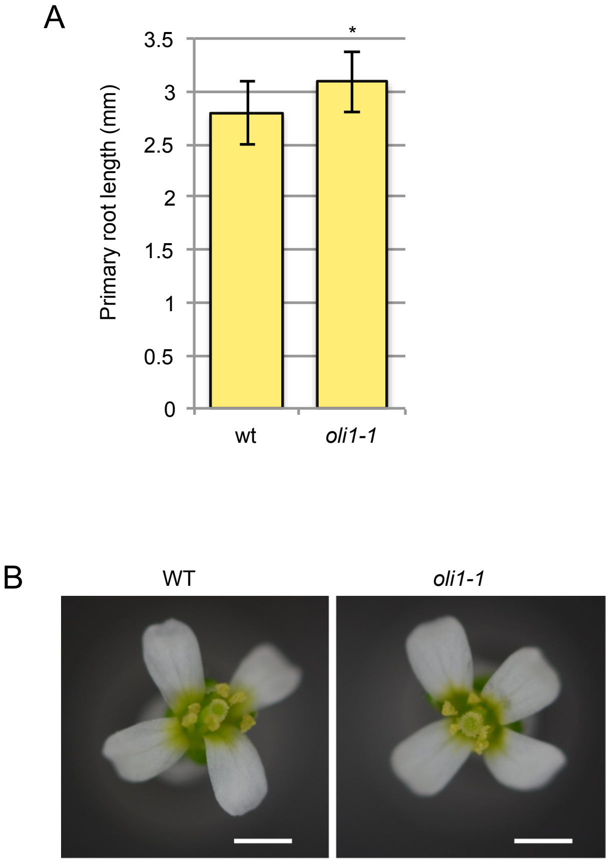

Fig. S1. Root and flower phenotypes in *oli1-1*.

Primary root lengths of 10-day-old seedlings (A) and fully opened flowers (B) are shown. In (A), data are means ± s.d. ($n \geq 10$). An asterisk indicates a significant difference compared with the WT value (Student's *t*-test; $p < 0.05$). Bars in (B) indicate 1 mm.
