# Supplementary material for: OLIGOCELLULA1/HIGH EXPRESSION OF OSMOTICALLY RESPONSIVE GENES15 Promotes Cell Proliferation With HISTONE DEACETYLASE9 and POWERDRESS During Leaf Development in Arabidopsis thaliana
Source: Front Plant Sci. 2018 May 3;9:580. doi: 10.3389/fpls.2018.00580 (PMC5943563; doi:10.3389/fpls.2018.00580)
Supplement: Supplementary file 7 [file Presentation_2.PDF]

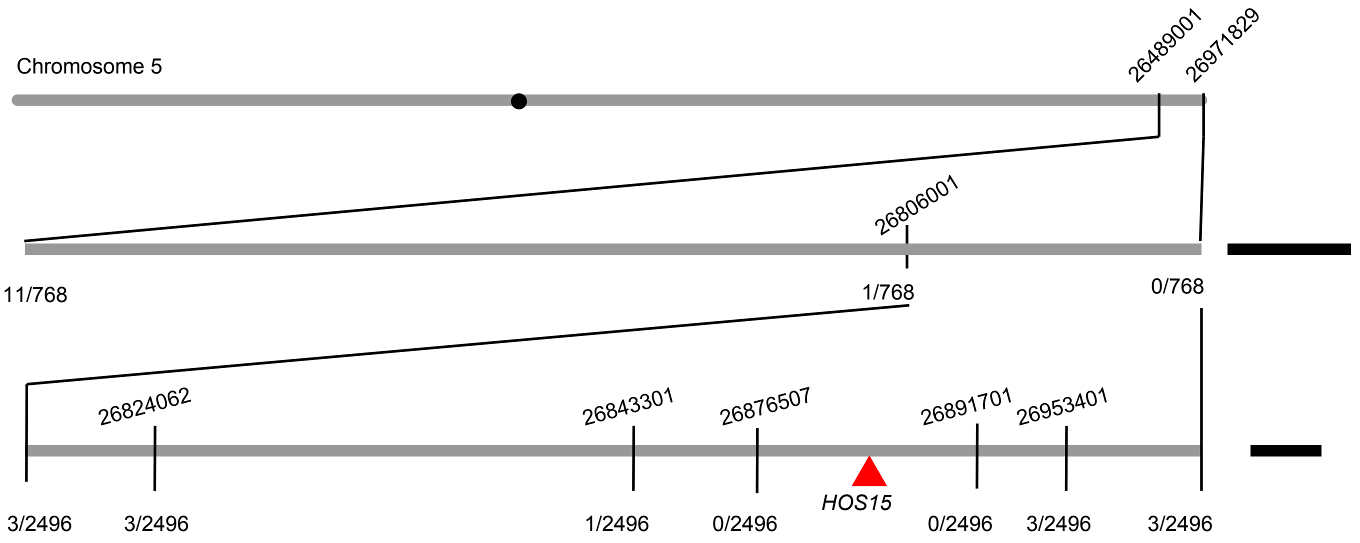

Fig. S2. Genetic mapping of the *oli1-1* mutation.

Chromosome 5 is shown schematically in the upper row. Enlarged parts of the lower end of chromosome 5 are shown in the middle and bottom rows. Gray bars indicate chromosomal DNA. Numbers on the chromosome indicate the position of polymorphic markers used in the genetic mapping. Numbers below the chromosome indicate the number of recombinants/total chromosomes analyzed for each polymorphic marker. The position of the *HOS15* locus is indicated by a red triangle. Black bars in the middle and bottom rows indicate 50 and 10 kb, respectively.
