# Supplementary material for: OLIGOCELLULA1/HIGH EXPRESSION OF OSMOTICALLY RESPONSIVE GENES15 Promotes Cell Proliferation With HISTONE DEACETYLASE9 and POWERDRESS During Leaf Development in Arabidopsis thaliana
Source: Front Plant Sci. 2018 May 3;9:580. doi: 10.3389/fpls.2018.00580 (PMC5943563; doi:10.3389/fpls.2018.00580)
Supplement: Supplementary file 8 [file Presentation_3.PDF]

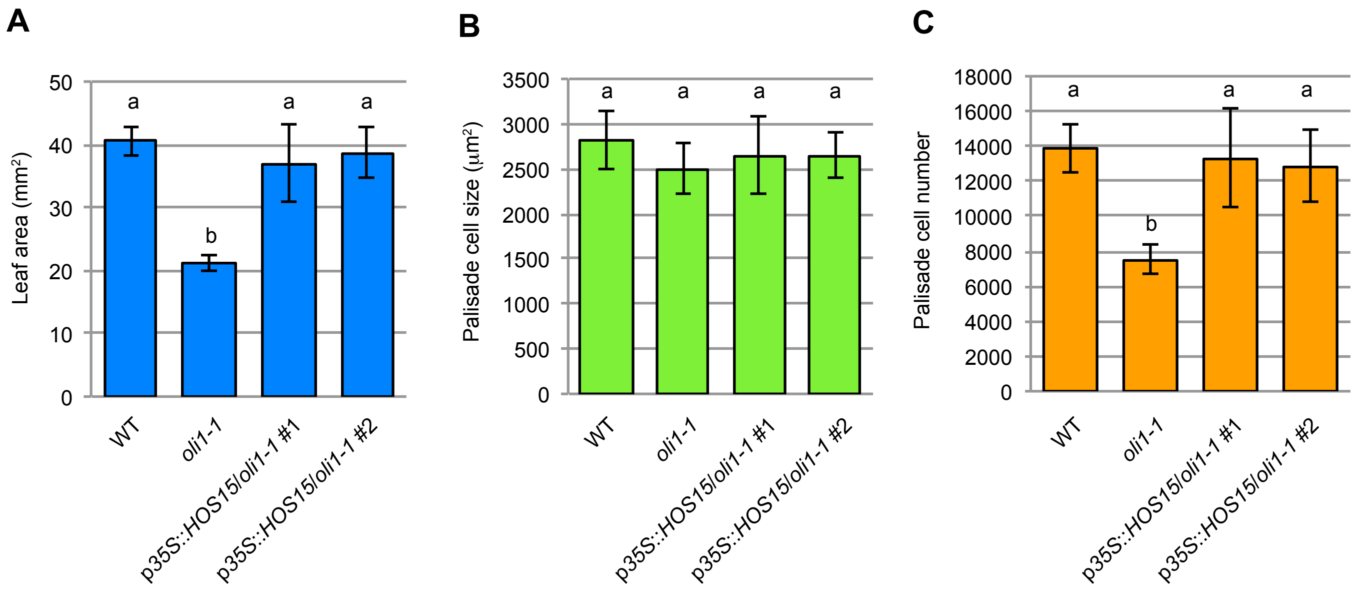

Fig. S3. Complementation tests of *oli1-1* by a p*35S*::*HOS15* construct.
(A) Areas of first leaves. (B) Palisade cell area. (C) Estimated palisade cell numbers. The first leaves were harvested from 25-day-old seedlings. Quantitative data are shown as means ± s.d. (n = 15 or 16). Statistical analysis was carried out using one-way ANOVA followed by Tukey-Kramer post-hoc test ($p < 0.05$). Statistically insignificant data are labeled by the same letter.
