# Supplementary material for: OLIGOCELLULA1/HIGH EXPRESSION OF OSMOTICALLY RESPONSIVE GENES15 Promotes Cell Proliferation With HISTONE DEACETYLASE9 and POWERDRESS During Leaf Development in Arabidopsis thaliana
Source: Front Plant Sci. 2018 May 3;9:580. doi: 10.3389/fpls.2018.00580 (PMC5943563; doi:10.3389/fpls.2018.00580)
Supplement: Supplementary file 9 [file Presentation_4.PDF]

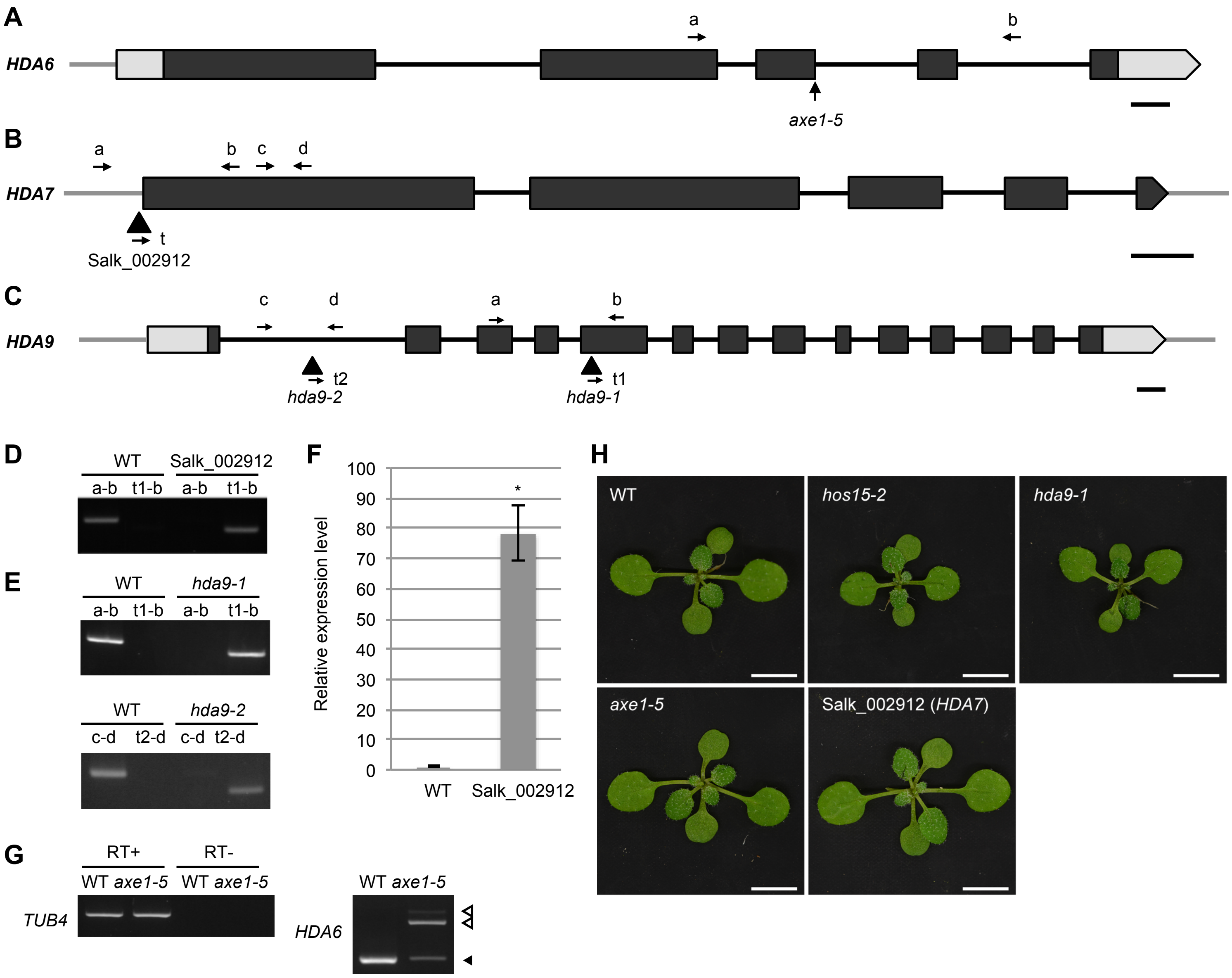

Fig. S4. Shoot phenotypes of Rpd3-type class I HDAC mutants.
(A) The mutation point of *axe1-5*. A primer pair used in RT-PCR is indicated by arrows. (B) and (C) T-DNA insertion sites of *hda7* and *hda9* alleles. T-DNA insertions are shown by triangles. Arrows indicate approximate primer positions used in genotyping or RT-qPCR. Bars, 100 bp. (D) and (E) Genotyping of *hda7* (D) and *hda9* (E) alleles. Primer pairs are indicated by letters shown in (B) and (C). (F) Relative expression levels of *HDA7* in WT and Salk_002912 determined by RT-qPCR using the primer pair (c and d) shown in (B) . n = 3, mean ± s.d. An asterisk indicates a significant difference from the WT value (Student's *t*-test; $p < 0.05$). (G) RT-PCR analysis of *HDA6* transcripts in WT and *axe1-5*. RT+ and RT- indicate that PCR was carried out using templates prepared with or without reverse transcription. PCR products amplified from normally and abnormally spliced *HDA6* transcripts are indicated by filled and open triangles, respectively. (H) Seedlings of WT and HDAC mutants grown for 15 days. Bars, 5 mm.
