# Supplementary material for: OLIGOCELLULA1/HIGH EXPRESSION OF OSMOTICALLY RESPONSIVE GENES15 Promotes Cell Proliferation With HISTONE DEACETYLASE9 and POWERDRESS During Leaf Development in Arabidopsis thaliana
Source: Front Plant Sci. 2018 May 3;9:580. doi: 10.3389/fpls.2018.00580 (PMC5943563; doi:10.3389/fpls.2018.00580)
Supplement: Supplementary file 10 [file Presentation_5.PDF]

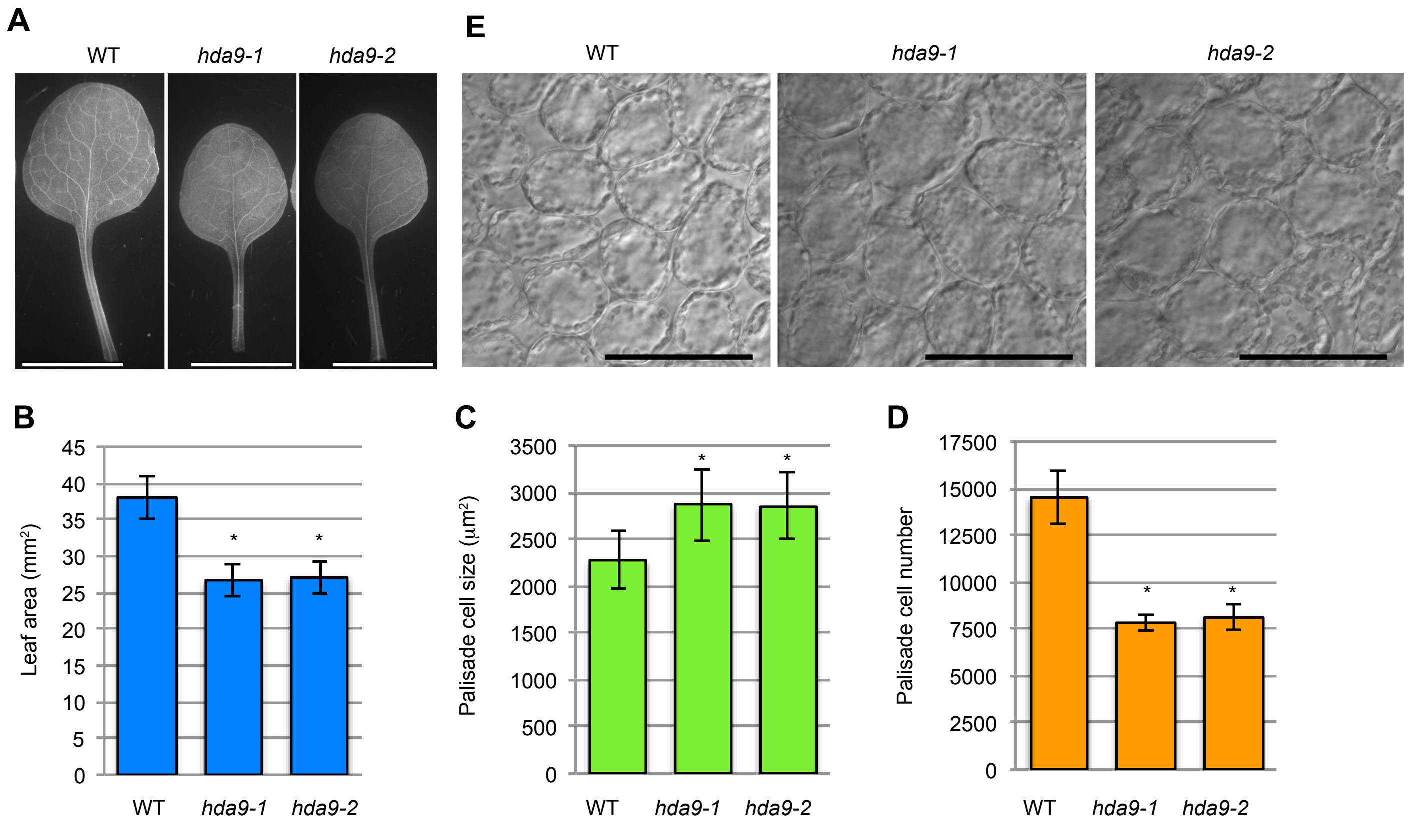

Fig. S5. Characterization of the leaf phenotypes in *hda9* alleles.

(A) First leaves. (B) Areas of first leaves. (C) Palisade cell area. (D) Estimated palisade cell numbers. (E) Palisade cells observed from the paradermal view. Bars in (A) and (E) indicate 5 mm and 100 μm, respectively. The first leaves were harvested from 25-day-old seedlings. Quantitative data are shown as means ± s.d. (n = 10), and asterisks indicate significant differences compared with the WT values (Student's *t*-test; $p < 0.05$).
