# Supplementary material for: OLIGOCELLULA1/HIGH EXPRESSION OF OSMOTICALLY RESPONSIVE GENES15 Promotes Cell Proliferation With HISTONE DEACETYLASE9 and POWERDRESS During Leaf Development in Arabidopsis thaliana
Source: Front Plant Sci. 2018 May 3;9:580. doi: 10.3389/fpls.2018.00580 (PMC5943563; doi:10.3389/fpls.2018.00580)
Supplement: Supplementary file 11 [file Presentation_6.PDF]

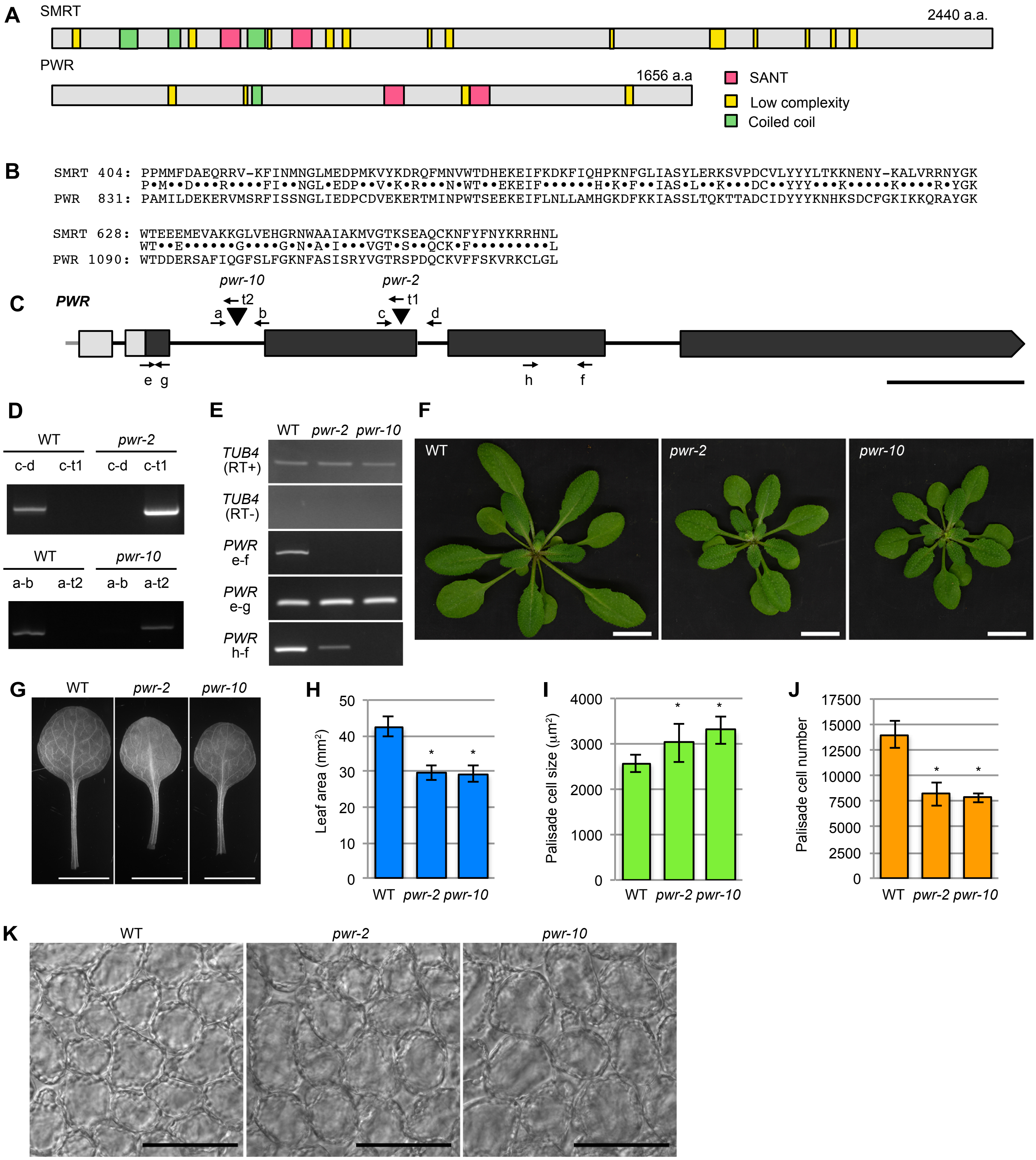

Fig. S6. Characterization of *pwr* alleles.

(A) Schematic diagrams of SMRT and PWR proteins. (B) Alignments of SANT domains of SMRT and PWR. Nonconserved amino acid residues are indicated by dots. (C) T-DNA insertion sites of *pwr* alleles. Arrows indicate the approximate positions of primers used in genotyping and RT-PCR analysis. (D) Genotyping of *pwr* alleles. Letters indicate primer pairs indicated in (C). (E) RT-PCR analysis of *TUB4* and *PWR*. 'RT +' and 'RT –' indicate that the templates used in PCR were prepared with (RT +) or without (RT –) reverse transcription, respectively. (F) Shoots of 25-day-old plants. (G) First leaves. (H) Areas of first leaves. (I) Palisade cell area. (J) Estimated palisade cell numbers. (K) Palisade cells observed from the paradermal view. Bar in (C) indicates 1 kb. Bars in (F), (G), and (K) indicate 1 cm, 5 mm, and 100 µm, respectively. The first leaves were harvested from 25-day-old seedlings. Quantitative data are shown as means ± s.d. ($n = 10$), and asterisks indicate significant differences compared with the WT values (Student's *t*-test; $p < 0.05$).
