# Supplementary figures and images for: OLIGOCELLULA1/HIGH EXPRESSION OF OSMOTICALLY RESPONSIVE GENES15 Promotes Cell Proliferation With HISTONE DEACETYLASE9 and POWERDRESS During Leaf Development in Arabidopsis thaliana
Source: Front Plant Sci. 2018 May 3;9:580. doi: 10.3389/fpls.2018.00580 (PMC5943563; doi:10.3389/fpls.2018.00580)

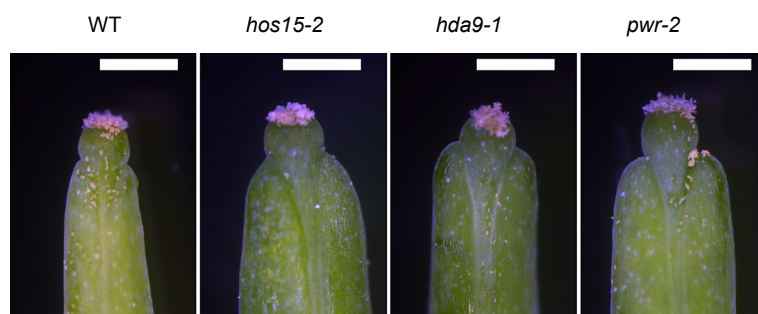

**Fig. S7. Fruit shape phenotypes.**  
Bars indicate 0.5 mm.

Supplement: Supplementary file 12 [file Presentation_7.PDF]
