# Supplementary material for: OLIGOCELLULA1/HIGH EXPRESSION OF OSMOTICALLY RESPONSIVE GENES15 Promotes Cell Proliferation With HISTONE DEACETYLASE9 and POWERDRESS During Leaf Development in Arabidopsis thaliana
Source: Front Plant Sci. 2018 May 3;9:580. doi: 10.3389/fpls.2018.00580 (PMC5943563; doi:10.3389/fpls.2018.00580)
Supplement: Supplementary file 13 [file Presentation_8.PDF]

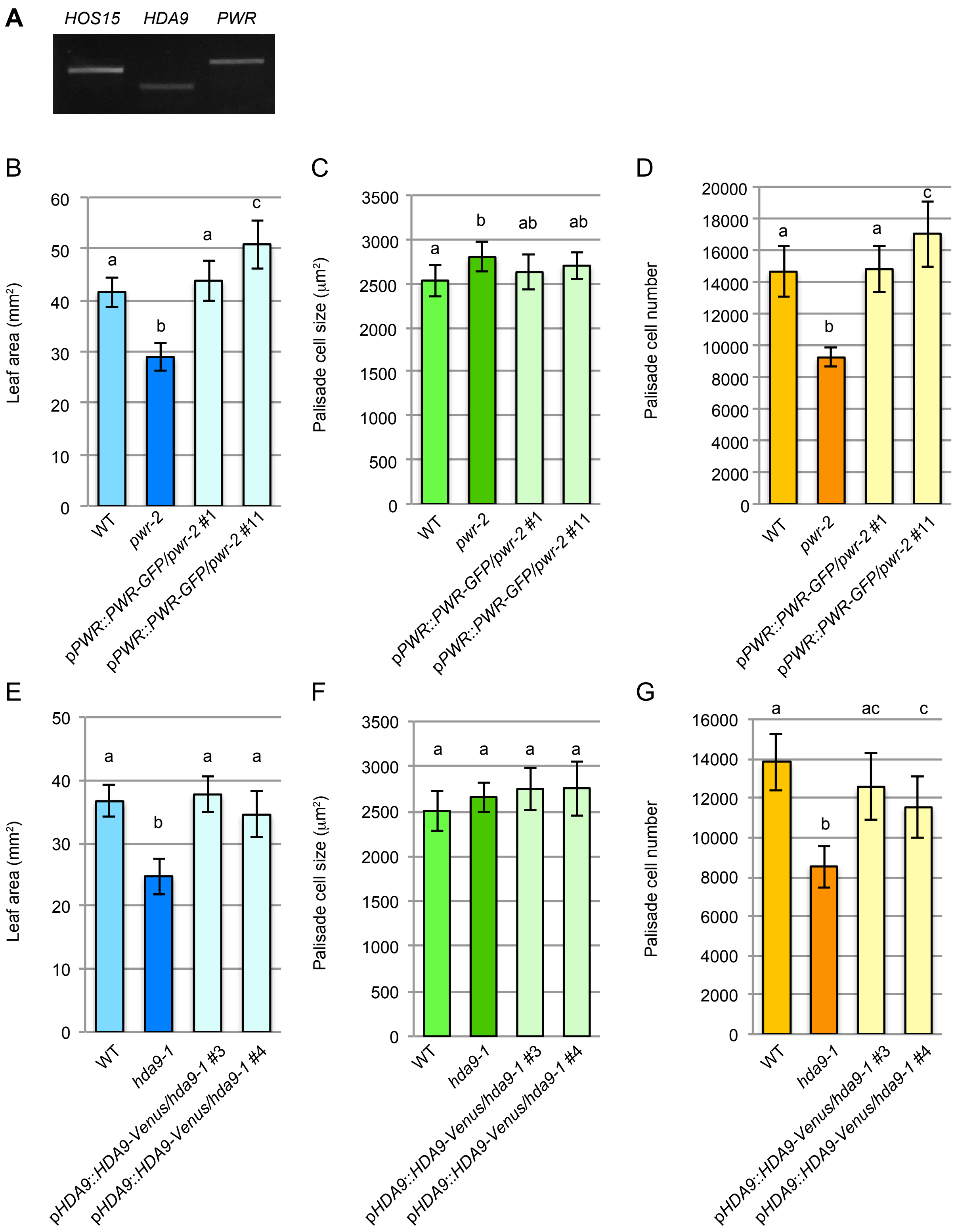

Fig. S8. Phenotypic rescue of *pwr-2* and *hda9-1* by PWR-GFP and HDA9-Venus constructs.

(A) RT-PCR analysis of *HOS15*, *HDA9*, and *PWR* using total RNAs isolated from 8-day-old first and second leaf primordia in WT. (B, E) Leaf blade area. (C, F) Palisade cell size. (D, G) Palisade cell number. In (B) to (D) WT, *pwr-2*, and transgenic *pwr-2* plants carrying a p*PWR*::*PWR-GFP* construct (#1 and #11) were examined. In (E) to (G) WT, *hda9-1*, and transgenic *hda9-1* plants carrying a p*HDA9*::*HDA9-Venus* construct (#3 and #4) were examined. In (B) to (G) seedlings were grown for 21 days, and their first leaves were examined. n = 10 to 11 (B to D) and 9 to 10 (E to G). Data are means ± s.d. Statistical analysis was carried out using one-way ANOVA followed by Tukey-Kramer post-hoc test ($p < 0.05$). Statistically insignificant data are labeled by the same letter.
