# Supplementary material for: OLIGOCELLULA1/HIGH EXPRESSION OF OSMOTICALLY RESPONSIVE GENES15 Promotes Cell Proliferation With HISTONE DEACETYLASE9 and POWERDRESS During Leaf Development in Arabidopsis thaliana
Source: Front Plant Sci. 2018 May 3;9:580. doi: 10.3389/fpls.2018.00580 (PMC5943563; doi:10.3389/fpls.2018.00580)
Supplement: Supplementary file 14 [file Presentation_9.PDF]

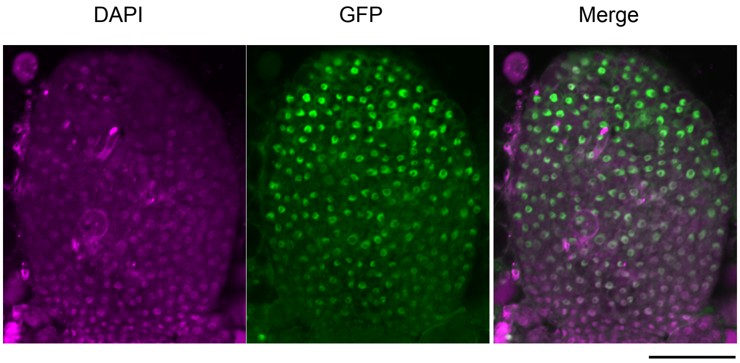

Fig. S9. Nuclear localization of GFP-HOS15.

A first leaf primoridum of a *GFP-HOS15* overexpression line. Scale bar indicates 50 μm.
