# Supplementary material for: OLIGOCELLULA1/HIGH EXPRESSION OF OSMOTICALLY RESPONSIVE GENES15 Promotes Cell Proliferation With HISTONE DEACETYLASE9 and POWERDRESS During Leaf Development in Arabidopsis thaliana
Source: Front Plant Sci. 2018 May 3;9:580. doi: 10.3389/fpls.2018.00580 (PMC5943563; doi:10.3389/fpls.2018.00580)
Supplement: Supplementary file 15 [file Presentation_10.PDF]

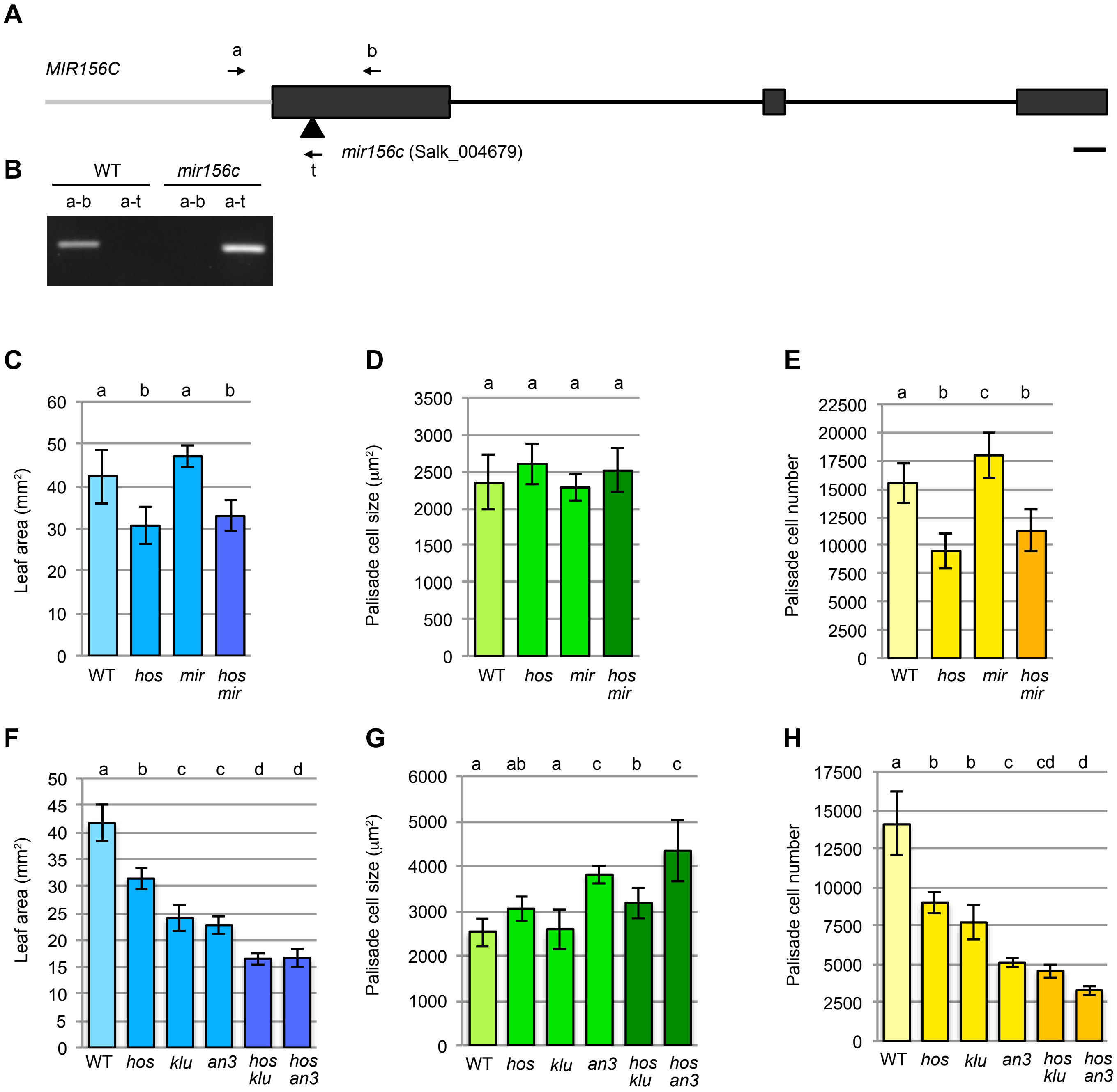

Fig. S10. Double mutant analyses between *hos15-2* and known leaf-size mutants.

(A) T-DNA insertion site of the *mir156c* mutant. Exons and introns are shown in black boxes and black lines, respectively. The promoter region of *MIR156C* is shown by a gray line. T-DNA insertion is shown by a triangle. Arrows indicate the approximate positions of primers used in genotyping. Bar, 100 bp. (B) Genotyping of *mir156c*. Letters indicate the primers shown in (A). (C) to (E) Double-mutant analyses between *hos15-2* and *mir156c*. (F) to (G) Double mutant analyses between *hos15-2* and *klu-4* or *an3-4*. (C) and (F) Leaf area. (D) and (G) Palisade cell size. (E) and (H) Palisade cell number. In (C) to (H), first leaves were harvested from 25-day-old plants, and the data are shown as means ± s.d. n = 10 in (C) to (E). n = 9 to 10 in (F) to (H). Statistical analyses in (C) to (E) and (F) to (H) are one-way ANOVA with Tukey's HSD test and the Tukey-Kramer test, respectively ($p < 0.05$).
