# Supplementary material for: OLIGOCELLULA1/HIGH EXPRESSION OF OSMOTICALLY RESPONSIVE GENES15 Promotes Cell Proliferation With HISTONE DEACETYLASE9 and POWERDRESS During Leaf Development in Arabidopsis thaliana
Source: Front Plant Sci. 2018 May 3;9:580. doi: 10.3389/fpls.2018.00580 (PMC5943563; doi:10.3389/fpls.2018.00580)
Supplement: Supplementary file 16 [file Presentation_11.PDF]

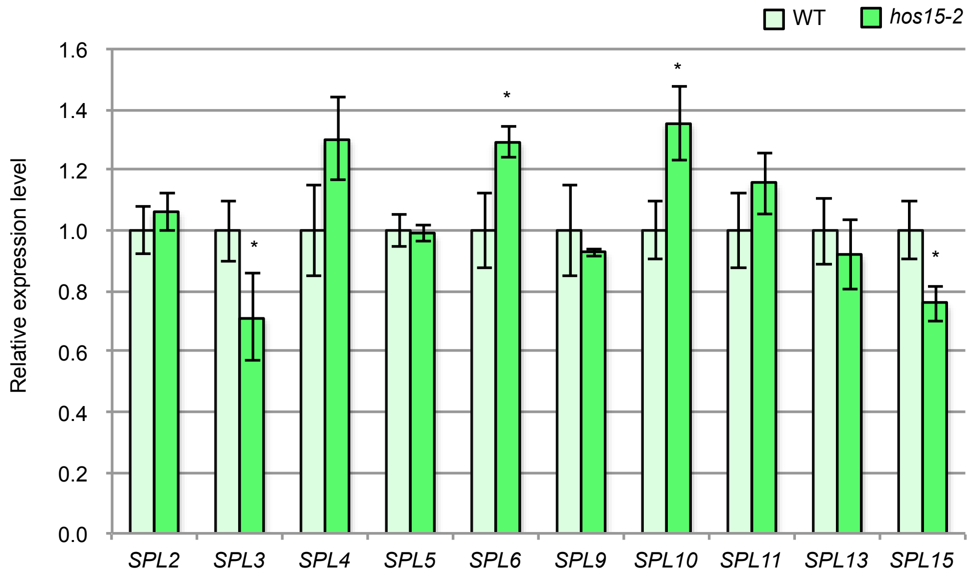

Fig. S11. Expression analyses of miR156-targeted *SPL* genes.

Expression levels of *SPL* genes in wild type and *hos15-2* were determined by RT-qPCR using 8d-old first pair of leaf primordia (n = 3, mean ± s.d.). Asterisks indicate significant differences compared with the WT values (Student's *t*-test; $p < 0.05$).
